# Anti‐tumor effects of PIM/PI3K/mTOR triple kinase inhibitor IBL‐302 in neuroblastoma

**DOI:** 10.15252/emmm.201911749

**Published:** 2020-01-09

**Authors:** Sofie Mohlin, Karin Hansson, Katarzyna Radke, Sonia Martinez, Carmen Blanco‐Aparicio, Cristian Garcia‐Ruiz, Charlotte Welinder, Javanshir Esfandyari, Michael O'Neill, Joaquin Pastor, Kristoffer von Stedingk, Daniel Bexell

The authors regret making a spelling error in Carmen Blanco‐Aparicio's name. The spelling is herewith corrected.

