# Supplementary material for: Anti‐tumor effects of PIM/PI3K/mTOR triple kinase inhibitor IBL‐302 in neuroblastoma
Source: EMBO Mol Med. 2019 Jul 16;11(8):e10058. doi: 10.15252/emmm.201810058 (PMC6685085; doi:10.15252/emmm.201810058)
Supplement: Supplementary file 1 — Appendix [file EMMM-11-e10058-s001.pdf]

EMM-2018-10058_Mohlin et al

Appendix

Table of Contents

Appendix Fig S1.

Relates to Fig. 2.

Appendix Fig S2.

Relates to Fig. 4.

Appendix Fig S3.

Relates to Fig. 6.

Appendix Table S1. *Overview of PDX characteristics.*

Appendix Table S2. *IBL-202 and IBL-301 GI_{50} ranges.*

Appendix Table S3. *Primer sequences used for quantitative RT-PCR.*

Appendix Table S4. *Detailed information on antibodies used for western blot and immunohistochemistry.*

Appendix Supplementary Methods. *Generation of compounds and Mass spectrometry.*

Appendix Supplementary Materials. *Raw screening data.*

Appendix Figure Legends

Appendix Fig S1. *PIM isoforms are expressed in neuroblastoma PDX cells.* **A-B**. *PIM1* (**A**) and *PIM3* (**B**) relative mRNA expression in LU-NB-2 and LU-NB-3 PDX cells and conventional cell line SK-N-BE(2)c determined by qRT-PCR. Data represents mean of n=3 biologically independent experiments and error bars represent SEM. **C-D.** Overall survival data for *PIM1* (**C**) and *PIM3* (**D**) in the publicly available datasets SEQC498 and Versteeg88 (R2: microarray analysis and visualization platform; http://r2.amc.nl). **E-I**. Sensitivity towards designated PI3K only inhibitors in cell lines from various tumor types as displayed by GI_{50}. Arrows indicate box plot for neuroblastoma.

Appendix Fig S2. *Treatment with IBL-302 induces differentiation and downregulates N-Myc.* **A-B.** Western blot analyses of downstream signaling protein (pAkt(Ser473) and pAkt(Thr308)) expression in LU-NB-2 cells after treatment with IBL-302 at indicated concentrations for 48 hours. Total Akt and Actin levels were used as loading controls. **C.** Brightfield photomicrographs of SK-N-SH cells treated with 50nM IBL-302 for 48 hours and quantification of neurite outgrowth presented as number of neurites. Representative areas (n=4 and n=7 for CTRL and IBL-302 respectively) were used and n=135 and n=144 cells/condition for CTRL and IBL-301, respectively, were counted. Statistical significance was determined by two-sided student's t-test. p=0.03. Scale bars represent 100µm. **D.** Weight of individual mice in control (n=5) or IBL-302 (n=5) treated groups over the course of 35 days (or until the mice were sacrificed due to tumor burden). **E.** Tumor size correlating to Fig. 5H.

Appendix Fig. S3. *IBL-302 potentiates the effect of doxorubicin and etoposide.* **A-B.** LU-NB-3 PDX cells were treated with indicated concentrations of chemotherapy (doxorubicin (**A**) or etoposide (**B**), respectively) and/or 50nM IBL-302 for 48 hours. Graphs show mean values and SEM from three independent experiments. **C.** Weight of individual mice in control (n=5), IBL-302 (n=5), cisplatin (n=5) or combination (n=5) treated groups over the course of 70 days (or until the mice were sacrificed due to tumor burden).

Appendix Table S1. Overview of PDX characteristics. *AG*; adrenal gland, *UD*; undifferentiated, *PD*; poorly differentiated.

Appendix Table S2. IBL-202, IBL-301 and IBL-302 GI_{50} ranges.

Appendix Table S3. Primer sequences used for quantitative RT-PCR.

Appendix Table S4. Detailed information on antibodies used for western blot, immunohistochemistry and immunofluorescence.

Appendix Supplementary Methods. Detailed generation of compounds and mass spectrometry methods.

Appendix Supplementary Materials. Raw data from IBL-302 screening across 707 cell lines from 47 tumor forms and a separate panel of 16 neuroblastoma cell lines.

Appendix Supplementary Methods

Generation of compounds

General Procedures: Chemicals were purchases from Aldrich Chemical Company Ltd., Apollo Scientific Ltd and TCI Europe N.V. Unless otherwise stated, commercial chemicals and solvents were used without further purification. Anhydrous DMF, DCM, toluene, MeOH, dioxane, acetonitrile, DIPEA and DME were purchased from Aldrich in Sure Seal™ bottle and kept under nitrogen. Proton (^{1}H) NMR spectra were recorded on Bruker Avance II 300 MHz spectrometer using DMSO-d_6, $CDCl_3$, CD_3OD as solvent. Chemical shifts are expressed in parts per million (ppm) (δ relative to residual solvent peak for ^{1}H). Multiplicities are indicated by s (singlet), d (doublet), t (triplet), q (quartet), m (multiplet), br (broad) or combination thereof. The HPLC measurements were performed using a HP 1100 from Agilent Technologies comprising a pump (binary) with degasser, an autosampler, a column oven, a diode-array detector (DAD) and a column Gemini-NX C18 (100 x 2.0 mm; 5µm particle size). Eluent A, water with 0.1% formic acid; eluent B: acetonitrile with 0.1% formic acid. Gradient 5% to 100% of B within 8 min at 50 ºC, DAD. Flow from the column was split to a MS spectrometer. The MS detector was configured with an electrospray ionization source or API/APCI. Nitrogen was used as the nebulizer gas. Data acquisition was performed with ChemStation LC/MSD quad, software. Purities of all reported compounds were greater than 95% based on HPLC chromatograms obtained on an Agilent HP 1100 LCMS system. All final compounds were purified to have purity higher than 95% by reverse phase high performance liquid chromatography (HPLC), or normal phase silica gel chromatography flash chromatography employing Biotage apparatus medium pressure liquid chromatography system SP4 or Isolera™ Prime, using ExtraBond Flash SLL with spacer SI (12 g, 20 g, 40 g, 80 g or 120 g). Thin layer chromatography (TLC) was performed on 20 mm precoated plates of silica gel (Merck, silica gel 60F254); visualization was achieved using ultraviolet light (254 nm).

Abbreviations: The term "BOC" means tert-butyloxycarbonyl protecting group; "DCM" means dichloromethane; "DCE" means dichloroethane; "MeOH" means methanol; "THF" means tetrahydrofuran; "DMF" means dimethylformamide; "DME" means 1,2-dimethoxyethane; "EtOAc" means ethyl acetate; "CH_3CN" means acetonitrile; "DIPEA" means diisopropylethylamine; "LDA" means lithium diisopropil amide; "TFA" means trifluoroacetic acid; "HATU" means O-(7-azabenzotriazole-1-yl)-1,1,3,3-tetramethyluronium hexafluorophosphate; "$PdCl_2(PPh_3)_2$" means Bis(triphenylphosphine)palladium(II) dichloride;

“HOAt” means 1-Hydroxy-7-azabenzotriazole; “RT” means room temperature; “min” means minutes; “sat.” means saturated; “aq.” means aqueous; eq. means equivalents.

General Synthetic Route to synthesize IBL-301, IBL-302 and IBL-202 (see also Figure 1A): **3-Formylamino-4-methyl-thiophene-2-carboxylic acid methyl ester (1):** Formic acid (24 mL) was added dropwise at 0ºC to acetic anhydride (36 mL). Methyl 3-amino-4-methylthiophene-2-carboxylate (10 g, 58.4 mmol) was added portion wise at 0ºC and the mixture was stirred at RT for 18 hours. The mixture was poured into a solution of Na_2CO_3 (60 g) in water (200 mL) cooled to 0ºC. The resulting white solid was then filtered off, washed with water and dried to give a white solid (**1**, 11 g), Y: 94%. ^{1}H NMR (300 MHz, DMSO) δ 9.85 (s, 1H), 8.24 (s, 1H), 7.55 (s, 1H), 3.76 (s, 3H), 2.07 (s, H). **7-Methyl-3H-thieno[3,2-d]pyrimidin-4-one (2):** A mixture of starting material **1** (11 g, 55.21 mmol, 1 eq.) and ammonium formate (27.85 g, 441.70 mmol, 8 eq.) in formamide (13 mL) was heated at 160ºC for 18 hours. On cooling to room temperature, water was added, and the resulting solid was filtered, washed off with water and dried to yield compound **2**, as a white solid (7.06 g, 77%). ^{1}H NMR (300 MHz, DMSO) δ 8.18 (s, 1H), 7.81 (s, 1H), 2.31 (s, 3H). **4-Chloro-7-methylthieno[3,2-d]pyrimidine (3):** A mixture of **2**, 10.2 g and $POCl_3$ (50 mL) was refluxed for 3 hours. Upon cooling, the solvents were removed in vacuo, and the residue suspended in cold water. Aqueous saturated Na_2CO_3 was added up to pH~8 at 0ºC. The resulting solid was filtered off, washed with water and dried, yielding compound **3**: 10.69 g, Y: 94%. ^{1}H NMR (300 MHz, DMSO) δ 9.01 (s, 1H), 8.19 (q, J = 1.1 Hz, 1H), 2.39 (d, J = 1.1 Hz, 3H). **4-Chloro-6-iodo-7-methyl-thieno[3,2-d]pyrimidine (4):** LDA (1.8 M in tetrahydrofuran, 38.45 ml, 69.189 mmol) was added to a mixture of **3** (8.87 g, 48.048 mmol) in THF (320 ml) at -78ºC. The mixture was stirred at -78ºC for 2 hours, then a solution of I_2 (18.30 g, 72.072 mmol) in THF (44.40 ml) was added slowly, via canula. The reaction was stirred at -78ºC for 2 hours. EtOAc was added to the mixture at -78ºC followed by the addition of an aq. saturated solution of $Na_2S_2O_3$. Aq. layer was extracted with EtOAc. The combined organic layers were washed with brine, dried (Na_2SO_4), filtered and concentrated to obtain desired product **4** as a pale brown solid (17 g, quantitative). ^{1}H NMR (300 MHz, $CDCl_3$) δ 8.85 (s, 1H), 2.40 (s, 3H). **5-(4-Chloro-7-methyl-thieno[3,2-d]pyrimidin-6-yl)-2-methoxy-pyridin-3-ylamine (5):** The reaction was carried out in two batches (2 x 7.38 g). Sealed tubes charged with compound **4** (7.38 g, 19.885 mmol theoric) in 1,4-dioxane (155 ml), (5-amino-6-methoxypyridin-3-yl)boronic acid pinacol ester (5.97 g, 23.861 mmol), Na_2CO_3 2M (52 ml) and dichlorobis(triphenylphosphine)palladium(II) (1.41 g,

1.988 mmol) were heated at 100ºC for 1 hour. The reaction was concentrated and the crude from both batches was purified by Biotage Flash Chromatography (in silica gel) using Cyclohexane-EtOAc gradient from 0% to 20% to give desired compound **5** (7.99 g, 13.453 mmol). ^{1}H NMR (300 MHz, $CDCl_3$) δ 8.94 (s, 1H), 7.78 (s, 1H), 7.08 (s, 1H), 4.09 (s, 3H), 2.49 (s, 3H). **{5-[6-(5-Amino-6-methoxy-pyridin-3-yl)-7-methyl-thieno[3,2-d]pyrimidin-4-yl]-thiophen -2-ylmethyl}-carbamic acid tert-butyl ester (6):** A mixture of compound **5** (1.0 g, 3.22 mmol), 5-(boc-aminomethyl)thiophene-2-boronic acid (1.0 g, 3.82 mmol), saturated aqueous solution of sodium carbonate (5 mL) and $PdCl_2[PPh_3]_2$ (270 mg) in degassed DME (100 mL) was refluxed for 5 hours under nitrogen. On cooling to room temperature, the solvents were removed in vacuo to give a residue that was purified by column chromatography (cyclohexane/EtOAc 75 to 100% mixtures as eluents) to give desired product **6** as a yellow solid (1.465 g, 90% yield). ^{1}H NMR (300 MHz, DMSO) δ 9.14 (s, 1H), 7.93 (d, J = 3.8 Hz, 1H), 7.75 (d, J = 2.2 Hz, 1H), 7.63 (t, J = 5.6 Hz, 3H), 7.23 (d, J = 2.2 Hz, 1H), 7.17 (d, J = 3.8 Hz, 1H), 5.33 (s, 2H), 4.37 (d, J = 5.6 Hz, 2H), 3.96 (s, 3H), 2.52 (s, 3H), 1.41 (s, 9H). **3-(5-{4-[5-(tert-Butoxycarbonylamino-methyl)-thiophen-2-yl]-7-methyl-thieno[3,2-d] pyrimidin-6-yl}-2-methoxy-pyridin-3-ylsulfamoyl)-benzoic acid methyl ester (7):** The reaction was carried out in two batches of 5 g each. To a solution of starting material **6** (5 g, 10.339 mmol) in pyridine (73.0 ml) methyl 3-(chlorosulfonyl)benzoate (3.06 g, 12.407 mmol) was added at 0ºC. The reaction mixture was stirred for 1 hour at 0 ºC. The reaction was quenched with MeOH and stirred for 30 min at 0ºC. The solvent was evaporated and the crude from all batches was purified by Biotage Flash Chromatography (in silica gel) using Cyclohexane-EtOAc gradient from 0% to 100% to give desired product **7** (6.980 g, 46%). ^{1}H NMR (300 MHz, MeOD) δ 8.89 (s, 1H), 8.45 (s, 1H), 8.20 (d, J = 7.8 Hz, 1H), 8.09 – 8.00 (m, 2H), 7.95 (s, 1H), 7.76 (s, 1H), 7.69 – 7.50 (m, 2H), 7.14 (ddd, J = 20.9, 13.5, 5.5 Hz, 1H), 4.44 (s, 2H), 3.89 (s, 3H), 3.79 (s, 3H), 2.38 (s, 3H), 1.48 (s, 9H). **3-(5-{4-[5-(tert-Butoxycarbonylamino-methyl)-thiophen-2-yl]-7-methyl-thieno[3,2-d] pyrimidin-6-yl}-2-methoxy-pyridin-3-ylsulfamoyl)-benzoic acid (8):** The reaction was carried out in two batches of 3.20 g each. To a solution of starting material **7** (3.20 g, 4.695 mmol) in 1,4-dioxane (46 ml), water (23 ml) was added to RT lithium hydroxide monohydrate (2.0 g, 46.95 mmol). The reaction mixture was stirred for 1 hour. The reaction mixture was concentrated and the crude from both batches was purified by Biotage Flash Chromatography (silica gel) using Cyclohexane-EtOAc gradient from 0% - 100% then EtOAc-MeOH gradient from 5 % - 60 % to give desired compound **8** (5.79 g, 8.67 mmol, 90 %). ^{1}H NMR (300 MHz, DMSO) δ 9.12 (s, 1H), 8.35 (t, J = 1.6 Hz, 1H), 8.07 (d, J = 7.2 Hz,

1H), 7.97 (d, J = 7.9 Hz, 2H), 7.91 (d, J = 3.9 Hz, 1H), 7.62 (m, 4H), 7.20 (d, J = 3.9 Hz, 1H), 4.38 (d, J = 6.0 Hz, 2H), 3.78 (s, 3H), 2.36 (s, 3H), 1.41 (s, 9H). **5-{5-[4-(5-Aminomethyl-thiophen-2-yl)-7-methyl-thieno[3,2-d]pyrimidin-6-yl]-2-methoxy-pyridin-3-ylsulfamoyl}-2-methoxy-benzoic acid (9):** To a solution of **8** (5.79 g, 8.666 mmol) in DCM (70 ml) at 0 ºC TFA (102 ml) was added dropwise. The reaction mixture was stirred for 1 hour at RT. The reaction mixture was concentrated and co-evaporated with toluene (three times) yielding a yellow solid, compound **9** (10.94 g) which was used in the next step without further purification. **5-{5-[4-(5-tert-Butoxyarboxyaminomethyl-thiophen-2-yl)-7-methyl-thieno[3,2-d] pyrimidin-6-yl]-2-methoxy-pyridin-3-ylsulfamoyl}-2-fluoro-benzoic acid (10):** 5-(Chlorosulfonyl)-2-fluorobenzoic acid (366 mg, 1.55 mmol) was added portion wise to a solution of compound **6** (500 mg, 1.03 mmol) in pyridine (20 mL) cooled to 0ºC and the mixture was stirred for 24 hours at RT. The solvents were removed in vacuo and the residue was purified by column chromatograph (EtOAc/MeOH 0 to 30% as eluents) to give compound **10** as a yellow solid (210 mg). ^{1}H NMR (300 MHz, DMSO) δ 9.14 (s, 1H), 8.23 (d, J = 5.2 Hz, 1H), 8.14 (m, 1H), 7.93 (d, J = 3.8 Hz, 1H), 7.88 (m, 1H), 7.67 (s, 2H), 7.40 (s, 1H), 7.19 (d, J = 3.9 Hz, 1H), 4.38 (d, J = 6.2 Hz, 2H), 3.80 (s, 3H), 2.42 (s, 3H), 1.42 (s, 9H). **5-{5-[4-(5-Aminomethyl-thiophen-2-yl)-7-methyl-thieno[3,2-d]pyrimidin-6-yl]-2-methoxy-pyridin-3-ylsulfamoyl}-2-fluoro-benzoic acid (11):** Compound **10** (210 mg, 0.3 mmol) was resuspended in DCE (5 mL), TFA (5 mL) was added and the mixture cooled to 0ºC. The mixture was stirred for 4 hours at RT and the solvents were removed in vacuo to give compound **11** as a brown solid (300 mg). **7-Chloro-2-iodo-3-methyl-thieno[3,2-b]pyridine (12):** LDA (1.8 M in tetrahydrofurane 0.73 ml, 1.307 mmol) was added to a mixture of 7-chloro-3-methylthieno[3,2-b]pyridine (206 mg, 1.089 mmol) in THF (7.2 ml) at -78ºC. The mixture was stirred at -78ºC for 1 hour, then a solution of I_2 (345.0 mg, 1.361 mmol) in THF (1.0 ml) was added slowly. The reaction was stirred at -78ºC for 1 hour. EtOAc was added to the mixture at -78ºC followed by the addition of an aq. saturated solution of $Na_2S_2O_3$. Aq layer was extracted with EtOAc. The combined organic layers were washed with brine, dried (Na_2SO_4), filtered and concentrated. The residue was triturated from CH_3CN to give the desired product **12** as a pale-yellow solid (291 mg, 0.940 mmol, 86%). LCMS (ESI): m/z 309.9/311.9 $[M+ H]^+$, Rt: 4.91 min. **5-(7-Chloro-3-methyl-thieno[3,2-b]pyridin-2-yl)-2-methoxy-pyridin-3-ylamine (13):** A mixture of compound **12** (100 mg, 0.323 mmol), (5-amino-6-methoxypyridin-3-yl)boronic acid pinacol esther (97 mg, 0.388 mmol), Na_2CO_3 2M (0.84 ml), dichlorobis(triphenylphosphine)palladium(II) (23 mg, 0.032 mmol) in 1,4-dioxane (2.52 ml) was heated at 100ºC for 1 hour in a sealed tube. The solvent was concentrated in vacuo and the

crude was purified by Biotage Flash Chromatography (silica gel) using Cyclohexane-EtOAc gradient from 0% - 30% to give desired product **13** pure (83 mg, 0.271 mmol, 84 %). LCMS (ESI): m/z 306.7 $[M+ H]^+$, Rt: 4.62 min. **{5-[2-(5-Amino-6-methoxy-pyridin-3-yl)-3-methyl-thieno[3,2-b]pyridin-7-yl]-thiophen-2-ylmethyl}-carbamic acid tert-butyl ester (14):** The reaction was carried out in two batches. Sealed tube charged with product **13** (597 mg, 1.952 mmol) in dioxane (15.25 ml), 5-(BOC-aminomethyl)thiophene-2-boronic acid (614 mg, 2.343 mmol), Na_2CO_3 2M (5.10 ml) and dichlorobis(triphenylphosphine)palladium(II) (138 mg, 0.195 mmol) was heated at 100ºC for 1 hour. Excess of reagents were added til almost completion of the reaction. The reaction was concentrated and the crude from both batches was purified in Biotage chromatography (silica gel) cyclohexane-EtOAc, 0% - 50% to give 2.08 g of compound **14** with traces of triphenylphosphine oxide. LCMS (ESI): m/z 483.6 $[M+ H]^+$, Rt: 4.78 min. **3-(5-{7-[5-(tert-Butoxycarbonylamino-methyl)-thiophen-2-yl]-3-methyl-thieno[3,2-b] pyridin-2-yl}-2-methoxy-pyridin-3-ylsulfamoyl)-benzoic acid methyl ester (15):** To a solution of **14** (370 mg, 0.767 mmol) in pyridine (5.40 ml) methyl 3-(chlorosulfonyl)benzoate (216 mg, 0.920 mmol) was added at 0ºC. The reaction mixture was stirred for 1 hour at 0ºC. The reaction mixture was quenched with MeOH and stirred for 30 min at 0ºC. The solvent was evaporated, and the crude was purified by Biotage Flash Chromatography (silica gel) using Cyclohexane-EtOAc gradient from 0% - 60% to give desired product **15** (249 mg, 0.366 mmol, 48 %). LCMS (ESI): m/z 681.8 $[M+ H]^+$, Rt: 5.06 min. **3-(5-{7-[5-(tert-Butoxycarbonylamino-methyl)-thiophen-2-yl]-3-methyl-thieno[3,2-b]pyridin-2-yl}-2-methoxy-pyridin-3-ylsulfamoyl)-benzoic acid (16):** To a solution of **15** (249 mg, 0.366 mmol) in 1,4-dioxane (3.59 ml) water (1.78 ml) was added to room temperature lithium hydroxide monohydrate (156 mg, 3.657 mmol). The reaction mixture was stirred for 1 hour and excess of base was again added (156 mg), stirring at RT for additional 30 min. The reaction mixture was concentrated, and the crude was purified by Biotage Flash Chromatography (silica gel) using Cyclohexane-EtOAc gradient from 0% - 100% then EtOAc-MeOH gradient from 0% - 30 % to give compound **16** (199 mg, 0.298 mmol, 82 %). LCMS (ESI): m/z 667.8 $[M+ H]^+$, Rt: 4.79 min. **3-{5-[7-(5-Aminomethyl-thiophen-2-yl)-3-methyl-thieno[3,2-b]pyridin-2-yl]-2-methoxy-pyridin-3-ylsulfamoyl}-benzoic acid (17):** To a solution of **16** (598 mg, 0.897 mmol) in DCM (7.10 ml) TFA (10.50 ml) was added dropwise at 0ºC. The reaction mixture was stirred for 1 hour at RT. The reaction mixture was concentrated and co-evaporated with toluene yielding yellow solid crude which was used in the next step without further purification as compound **17**. A small portion of the compound was purified by HPLC yielding pure white solid **17**, ^{1}H NMR (300 MHz, DMSO) δ 8.73 (d, J = 5.1 Hz, 1H), 8.67 (s, 1H), 8.15

(m, 2H), 7.97 (d, J = 8.1 Hz, 1H), 7.93 (d, J = 3.8 Hz, 1H), 7.87 (d, J = 5.1 Hz, 1H), 7.59 (t, J = 7.7 Hz, 1H), 7.50 (d, J = 2.1 Hz, 1H), 7.39 (d, J = 3.7 Hz, 1H), 4.51 (s, 2H), 3.97 (s, 3H), 2.50 (s, 3H, under solvent signal). LCMS (ESI): m/z 567.1 $[M+ H]^+$, Rt: 3.27 min.

IBL-301: The reaction carried 18 batches of 730 mg each. Compound **9** (730 mg, 0.578 mmol theoric) was dissolved in DMF (20 ml) and DIPEA (0.93 ml, 5.351 mmol). The material was slowly added (syringe pump; 3.5 mL/h) to a solution of HATU (449 mg, 1.156 mmol) and HOAt (0.5 M in DMF, 2.31 ml, 1.156 mmol) in DMF (70 ml). After addition, the reaction was stirred for 10 hours, then evaporated and the crude was purified by Biotage Flash Chromatography (silica gel) using Cyclohexane-EtOAc gradient from 0% - 100% followed by EtOAc -MeOH gradient from 5% - 10% to yield a product that was filtered and washed with MeOH and CH_3CN to give pure compound **IBL-301** (511 mg, 0.930 mmol). ^1H NMR (300 MHz, DMSO) δ 10.79 (s, 1H), 9.89 (t, J = 5.5 Hz, 1H), 9.17 (s, 1H), 8.60 (s, 1H), 8.36 (s, 1H), 8.26 (d, J = 7.8 Hz, 1H), 8.19 (d, J = 7.8 Hz, 1H), 7.91 (d, J = 3.7 Hz, 1H), 7.85 (t, J = 7.6 Hz, 1H), 7.61 (s, 1H), 7.25 (d, J = 3.6 Hz, 1H), 4.83 (d, J = 4.8 Hz, 2H), 4.05 (s, 3H), 2.60 (s, 3H). LCMS (ESI): m/z 550.1 $[M+ H]^+$, Rt: 4.99 min. **IBL-302:** A mixture of compound **11** (300 mg, 0.43 mmol) and DIPEA (0.6 mL) in dry DMF (60 mL) was added dropwise at room temperature for a 1.5 hours period over a solution of 1-hydroxy-7-azabenzotriazole (0.6 M solution in DMF; 1.96 mL) and HATU (443 mg) in DMF (150 mL). The mixture was stirred for 24 hours at RT and the solvents removed in vacuo to give a residue that was triturated from water to give a brown solid that was further purified by column chromatography (DCM/MeOH 0 to 20% mixtures as eluents) to give a brown solid (53 mg) that was recrystallized from MeCN/water to give compound **IBL-302** as a pale brown solid (39 mg). ^1H NMR (300 MHz, DMSO) δ 10.80 (s, 1H), 9.58 (t, J = 5.2 Hz, 1H), 9.18 (s, 1H), 8.48 (dd, J = 6.4, 2.5 Hz, 1H), 8.37 (s, 1H), 8.32 (dd, J = 7.4, 3.0 Hz, 1H), 7.93 (d, J = 3.7 Hz, 1H), 7.77 (t, J = 9.5 Hz, 1H), 7.73 (d, J = 2.0 Hz, 1H), 7.24 (d, J = 3.7 Hz, 1H), 4.85 (d, J = 4.3 Hz, 2H), 4.05 (s, 3H), 2.61 (s, 3H). LCMS (ESI): m/z 568.1 $[M+ H]^+$, Rt: 5.21 min. **IBL-202:** Compound **17** (306 mg, 0.298 mmol theoric) was dissolved in DMF (12.38 ml) and DIPEA (0.40 ml, 2.251 mmol). The mixture was slowly added (syringe pump; 2 mL/h) to a solution of HATU (232 mg, 0.597 mmol) and HOAt (0.5 M in DMF, 1.19 ml, 0.597 mmol) in DMF (47.32 ml). After addition, the reaction was stirred for 10 hours, then evaporated and the resulting crude was purified by Biotage Flash Chromatography (silica gel) using Cyclohexane-EtOAc gradient from 0% - 100% to give expected product which was triturated with MeOH and CH_3CN to yield pure compound **IBL-202** (51 mg, 0.093 mmol). ^1H NMR (300 MHz, DMSO) δ 10.70 (s, 1H), 9.84

(t, J = 6.0 Hz, 1H), 8.71 (d, J = 5.0 Hz, 1H), 8.60 (s, 1H), 8.32 (d, J = 2.1 Hz, 1H), 8.28 (d, J = 8.8 Hz, 1H), 8.16 (d, J = 7.8 Hz, 1H), 7.84 (t, J = 7.8 Hz, 1H), 7.73 (d, J = 4.9 Hz, 1H), 7.67 (d, J = 3.6 Hz, 1H), 7.60 (d, J = 2.1 Hz, 1H), 7.20 (d, J = 3.6 Hz, 1H), 4.78 (d, J = 5.5 Hz, 2H), 4.04 (s, 3H), 2.62 (s, 3H). LCMS (ESI): m/z 549.1 $[M+ H]^+$, Rt: 4.80 min.

Mass spectrometry

Cell samples and protein extraction

Cells, 5 million, were lysed with 4 M urea in 100 mM ammonium bicarbonate (AMBIC) and mixed using a Vortex mixer then the samples were placed on ice for 15 min. The samples were mixed again and were placed on ice for another 15 min. The samples were placed in a Bioruptor® Plus (Diagenode) and the settings were: 50 cycles of 15 sec on and 15 sec off. The samples were centrifuged 10 000 x g for 10 min at 4 °C. The supernatants were collected and protein determination was done by MicroBCA™ Protein Assay Kit (Thermo Scientific) according to the manufacturer instructions.

In-solution digestion with Lys C and trypsin

Samples were diluted with 4 M urea in 100 mM AMBIC to a concentration of 2.5 µg/µL in a final volume of 40 µL. The digestion was performed using a Bravo robot (Agilent Technologies). Reduction was performed by adding 10 µL 70 mM dithiothreitol and incubated for 1 h at 37 °C followed by alkylation using 20 µL 70 mM iodoacetamide for 30 min at room temperature. Digestion was started by adding 90 µL 0.022 µg/µL LysC (Mass Spec Grade, Cat # VA1170, Promega). After 5 hrs at room temperature 108 µL 0.0185 µg/µL trypsin (Sequence Grade Modified #V511, Promega) was added and incubated 16 hrs at room temperature. The digestion was stopped by adding 12 µL 10% trifluoroacetic acid (TFA). Total sample volume 280 µL.

Peptide clean-up

Peptide clean-up was performed using the Bravo robot. AssayMAP cartridge Reverse Phase S (Cat # G5496-60033, Agilent Technologies) was used for the clean up to remove salts. The C18 cartridge was primed with 100 µL 0.1% TFA, 50% acetonitrile (ACN) in water followed by equilibration with 70 µL 0.1% TFA in water. The samples, 280 µL were loaded to the cartridge and washed with 50 µL 0.1% TFA in water three times. The peptides were eluted with 35 µL 80% ACN, 0.1% TFA in water. For the global analysis the samples were dried using a Speed Vac and resolved in 2% ACN, 0.1% TFA in water.

Phosphopeptides enrichment

Enrichment of phosphopeptides was performed using the Bravo robot. Before the enrichment the peptides had to be clean-up by the C18 cartridges, but were used directly without drying. For the enrichment of phosphopeptides AssayMAP cartridge Fe(III)-NTA (Cat # G5496-60085, Agilent Technologies) was used. The cartridge was primed with 100 µL 50% ACN, 0.1% TFA in water followed by equilibration with 50 µL 80%, 0.1% TFA in water. The samples, 280 µL were loaded to the cartridge and washed with 50 µL 80%, 0.1% TFA in water three times. The peptides were eluted with 25 µL 5% ammonium hydroxide and acidified with 10 µL 50% formic acid. The samples were dried using a Speed Vac and resolved in 2% CAN, 0.1% TFA in water.

Peptide determination

For the global analysis a peptide determination was performed before injection into the liquid chromatography mass spectrometer (LC-MS) using Pierce™Quantitative Colorimetric Peptide assay (Thermo Scientific) according to the manufacturer instructions.

Mass spectrometry acquisition

The LC-MS detection was performed on Tribrid mass spectrometer Fusion equipped with a Nanospray Flex ion source and coupled with an EASY-nLC 1000 ultrahigh pressure liquid chromatography (UHPLC) pump (Thermo Fischer Scientific). For the global analysis, 1 µg of the peptides was injected and for the phosphopeptide analysis the whole sample was injected into the LC-MS. Peptides were concentrated on an Acclaim PepMap 100 C18 precolumn (75 µm x 2 cm, Thermo Scientific, Waltham, MA) and then separated on an Acclaim PepMap RSLC column (75 µm x 25 cm, nanoViper, C18, 2 µm, 100 Å) at the temperature of 40 °C and with a flow rate of 300 nL/min. Solvent A (0.1% formic acid in water) and solvent B (0.1% formic acid in acetonitrile) were used to create a nonlinear gradient to elute the peptides. For the gradient, the percentage of solvent B was maintained at 3% for 3 min, increased from 3% to 30% for 90 min and then increased to 60% for 15 min and then increased to 90% for 5 min and then kept at 90% for another 7 min to wash the column.

The Orbitrap Fusion was operated in the positive data-dependent acquisition (DDA) mode. The peptides were introduced into the LC-MS via stainless steel Nano-bore emitter (OD 150 µm, ID 30 µm) with the spray voltage of 2 kV and the capillary temperature was set 275 °C. Full

MS survey scans from m/z 350-1350 with a resolution of 120,000 were performed in the Orbitrap detector. The automatic gain control (AGC) target was set to 4×10^5 with an injection time of 50 ms. The most intense ions (up to 20) with charge states 2-5 from the full scan MS were selected for fragmentation in the Orbitrap. The MS2 precursors were isolated with a quadrupole mass filter set to a width of 1.2 m/z. Precursors were fragmented by high-energy collision dissociation (HCD) at a normalized collision energy (NCE) of 30%. The resolution was fixed at 30000 and for the MS/MS scans, the values for the AGC target and injection time were 5×10^4 and 54 ms, respectively. The duration of dynamic exclusion was set to 45s and the mass tolerance window was 10 ppm.

Data analysis

The raw DDA data were analyzed with Proteome Discoverer™ 2.2 (PD 2.2) Software (Thermo Fisher Scientific). Peptides were identified using SEQUEST HT against UniProtKB human database (release 20171218). The search was performed with the following parameters applied: static modification: cysteine carbamidomethylation and dynamic modifications: N-terminal acetylation, methionine oxidation and phosphorylation. Precursor tolerance was set to 10 ppm and fragment tolerance was set to 0.05 ppm. Up to 2 missed cleavages were allowed and Percolator was used for peptide validation at a q-value of maximum 0.01. Filter settings at the protein level: Master is equal to Master, Protein Unique Peptides is greater than or equal to 2, Found in Files has confidence High in at least 2 files. Filter settings peptide level: Found in Samples has confidence High in at least 2 samples. Extracted peptides were used to identify and quantify them by label-free relative quantification. The extracted chromatographic areas were used to compare peptide abundance across samples.

Chemicals

Ammonium bicarbonate (A6141), urea (U1250), dithiothreitol (D0632), iodoacetamide (I-1149, trifluoroacetic acid (T6399) were purchased from Sigma-Aldrich. Acetonitrile (1.00029) and Water (1.15333) were purchased from LiChrosolv. Formic acid (A117-50) was purchased from Fisher Chemical. Ammonium hydroxide (3388-18) was purchased from Honeywell.

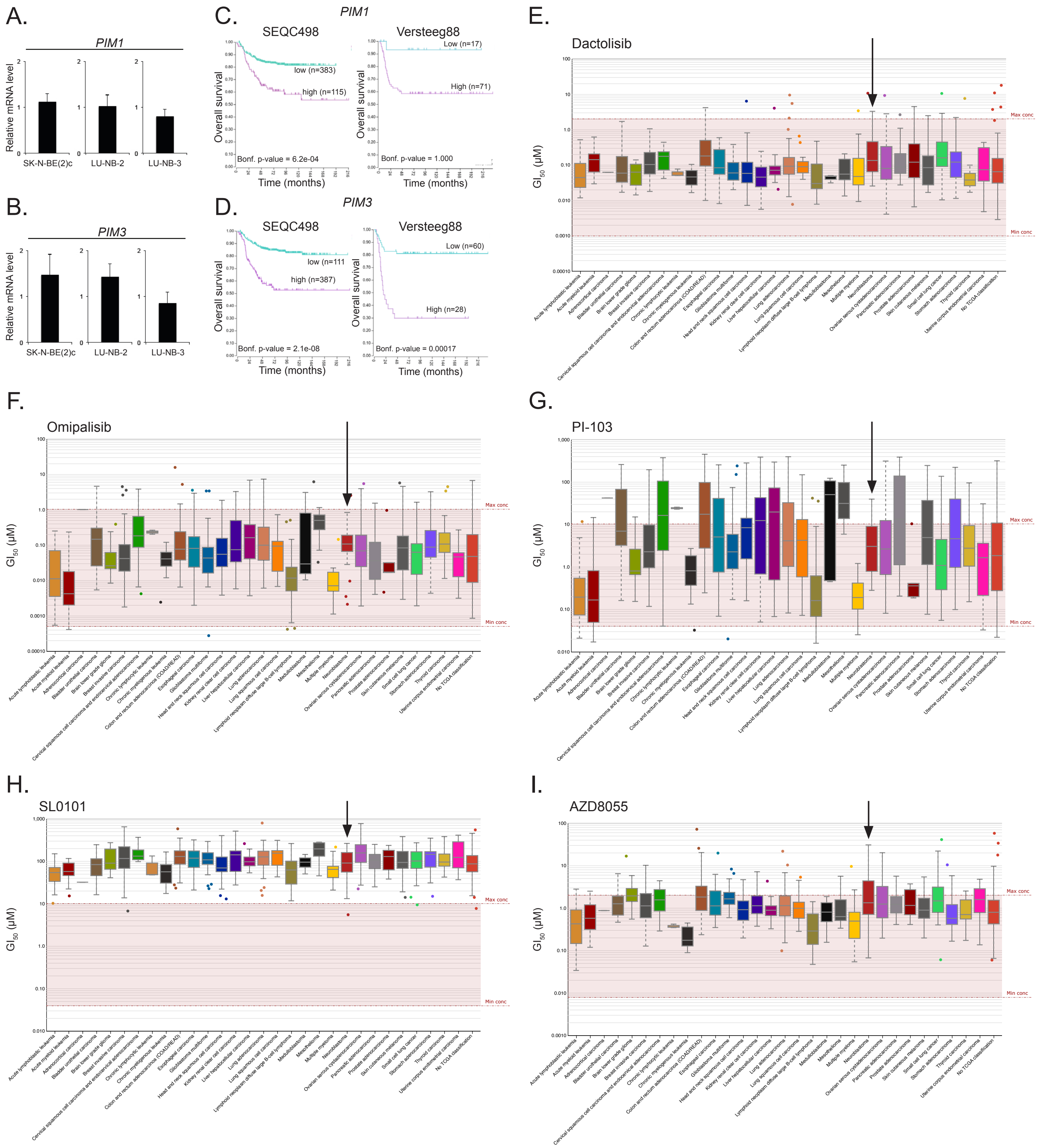
A.
PIM1
Relative mRNA level
SK-N-BE(2)c
LU-NB-2
LU-NB-3
B.
PIM3
Relative mRNA level
SK-N-BE(2)c
LU-NB-2
LU-NB-3
C.
PIM1
SEQC498
low (n=383)
high (n=115)
Bonf. p-value = 6.2e-04
Versteeg88
Low (n=17)
High (n=71)
Bonf. p-value = 1.000
Overall survival
Time (months)
D.
PIM3
SEQC498
low (n=111
high (n=387)
Bonf. p-value = 2.1e-08
Versteeg88
Low (n=60)
High (n=28)
Bonf. p-value = 0.00017
Overall survival
Time (months)
E.
Dactolisib
F.
Omipalisib
G.
PI-103
H.
SL0101
I.
AZD8055
GI_{50} (μM)
Max conc
Min conc

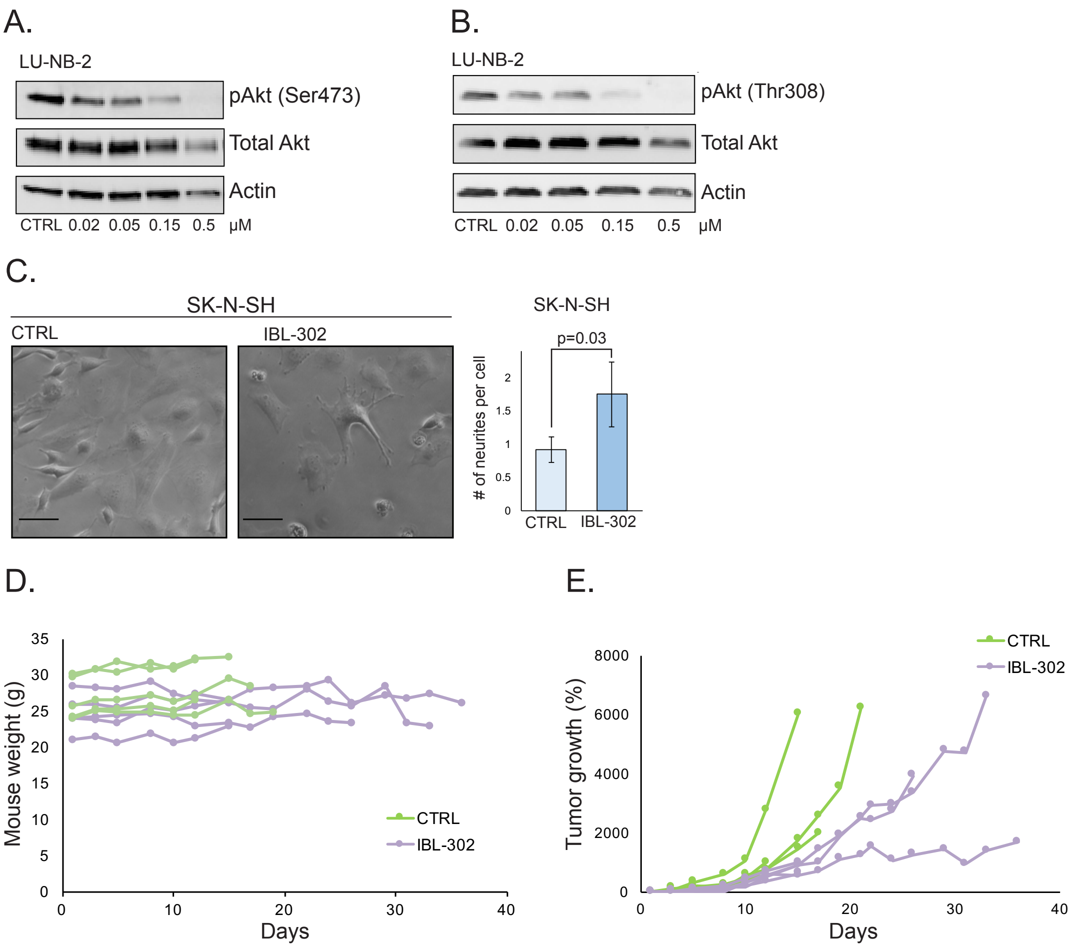
A.
LU-NB-2
pAkt (Ser473)
Total Akt
Actin
CTRL 0.02 0.05 0.15 0.5 µM
B.
LU-NB-2
pAkt (Thr308)
Total Akt
Actin
CTRL 0.02 0.05 0.15 0.5 µM
C.
SK-N-SH
CTRL
IBL-302
SK-N-SH
p=0.03
# of neurites per cell
0
0.5
1
1.5
2
CTRL
IBL-302
D.
Mouse weight (g)
0
5
10
15
20
25
30
35
0
10
20
30
40
Days
CTRL
IBL-302
E.
Tumor growth (%)
0
2000
4000
6000
8000
0
10
20
30
40
Days
CTRL
IBL-302

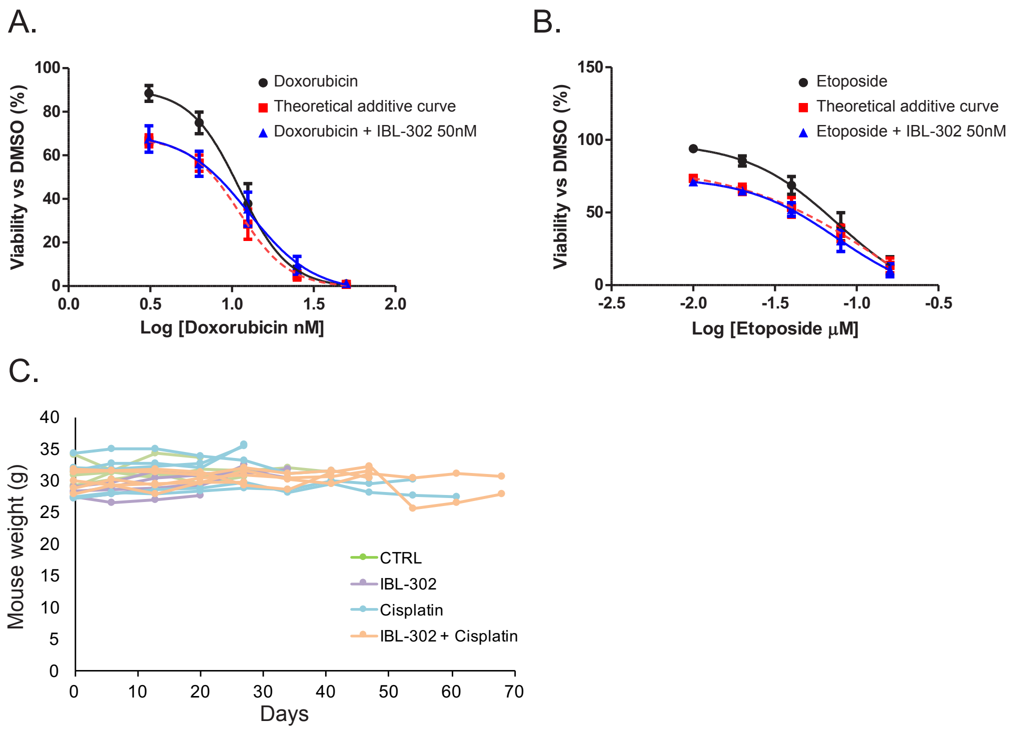

A.
100
80
60
40
20
0
Viability vs DMSO (%)
0.0
0.5
1.0
1.5
2.0
Log [Doxorubicin nM]
Doxorubicin
Theoretical additive curve
Doxorubicin + IBL-302 50nM
B.
150
100
50
0
Viability vs DMSO (%)
-2.5
-2.0
-1.5
-1.0
-0.5
Log [Etoposide µM]
Etoposide
Theoretical additive curve
Etoposide + IBL-302 50nM
C.
40
35
30
25
20
15
10
5
0
Mouse weight (g)
0
10
20
30
40
50
60
70
Days
CTRL
IBL-302
Cisplatin
IBL-302 + Cisplatin

Cells	Patient age	Sample type	Chemotherapy	Stage	Histology	SNP profile
LU-NB-2	2y2m	Metastasis	Yes	IV	UD	MYCN amp, 1p-, +17q
LU-NB-3	2y9m	Primary (AG)	No	III	PD	MYCN amp, 1p-, +17q

Inhibitor	Target	GI_{50} range
IBL-202	PIM/PI3K	0.1-1.5μM
IBL-301	PIM/PI3K/mTOR	0.05-0.85μM
IBL-302	PIM/PI3K/mTOR	0.05-0.85μM

**Target gene**	**5' - 3'**	
UBC (Reference gene)	Fwd	ATTTGGGTCGCGGTTCTTG
	Rev	TGCCTTGACATTCTCGATGGT
SDHA (Reference gene)	Fwd	TGGGAACAAGAGGGCATCTG
	Rev	CCACCACTGCATCAAATTCATG
YWHAZ (Reference gene)	Fwd	ACTTTTGGTACATTGTGGCTTCAA
	Rev	CCGCCAGGACAAACCAGTAT
PIM1	Fwd	TTGGTTCCCTTCCTATTCCA
	Rev	CACCCCACAAACAGAGCA
PIM3	Fwd	CGATGCTGCTCTCCAAGTTC
	Rev	TCTCGAAGCTCTCCTTGTCC
GAP43	Fwd	ACGACCAAAAGATTGAACAAGATG
	Rev	TCCACGGAAGCTAGCCTGAA
MYCN	Fwd	CGCAAAAGCCACCTCTCATTA
	Rev	TCCAGCAGATGCCACATAAGG

Western Blot antibodies				
**Primary Antibody**	**Species**	**Dilution**	**Source**	**Product #**
Phospho-Akt (Ser-473)	Rabbit	1:2000	Cell Signaling	4060
Phospho-Akt (Thr-308)	Rabbit	1:1000	Cell Signaling	2965
Total Akt	Mouse	1:2000	Cell Signaling	2920
Cleaved Caspase3	Rabbit	1:1000	Cell signaling	9662
Gap43	Rabbit	1:1000	Cell signaling	8945
N-Myc	Rabbit	1:5000	Novus	23960002
N-Myc	Rabbit	1:500	Santa cruz Biotech	Sc-791
Phospho-p70 S6 Kinase (Thr389)	Rabbit	1:1000	Cell signaling	9205
p70 S6 Kinase	Rabbit	1:1000	Cell Signaling	9202
Phospho-PRAS40 (T246)	Rabbit	1:1000	Abcam	Ab134084
PRAS40	Rabbit	1:2000	Abcam	Ab134158
Actin	Mouse	1:2000	MP biomedicals	691001
SDHA	Mouse	1:4000	Abcam	Ab14715
**Secondary Antibody**	**Species**	**Dilution**	**Source**	
Anti-Mouse	Sheep	1:5000	Invitrogen	62-6520
Anti-Rabbit	Goat	1:5000	Invitrogen	65-6120

Immunohistochemistry antibodies				
**Primary Antibody**	**Species**	**Dilution**	**Source**	**Product #**
N-Myc	Rabbit	1:300	Novus	23960002

Immunofluorescence antibodies				
**Primary Antibody**	**Species**	**Dilution**	**Source**	**Product #**
Tuj1	Mouse	1:500	Covance	MMS-435P
**Secondary Antibody**	**Species**	**Dilution**	**Source**	
Alexa Fluor 488 goat anti-mouse	Goat	1:200	Invitrogen	A11029

Screening IBL-302 across a panel of 707 cell lines from 47 tumor types

COSMIC	Cell_line	tissue	tissue2	IBL-302	IBL-302 IC50
906870	GDM-1	leukemia	acute_myeloid_leukaemia	-4,984151604	0,006845583
908451	NOMO-1	leukemia	acute_myeloid_leukaemia	-2,500154167	0,082072345
909253	P31-FUJ	leukemia	acute_myeloid_leukaemia	-2,490408127	0,082876136
907280	KMOE-2	leukemia	acute_myeloid_leukaemia	-2,266013077	0,1037249
908448	NKM-1	leukemia	acute_myeloid_leukaemia	-2,218806153	0,108738849
910947	OCI-AML2	leukemia	acute_myeloid_leukaemia	-2,082427444	0,124627319
908148	MONO-MAC-6	leukemia	acute_myeloid_leukaemia	-1,69795672	0,183057179
908141	ML-2	leukemia	acute_myeloid_leukaemia	-1,402887276	0,245885997
1330947	MOLM-13	leukemia	acute_myeloid_leukaemia	-1,396622607	0,247431227
905938	HL-60	leukemia	acute_myeloid_leukaemia	-1,339603526	0,261949504
1290455	OCI-AML3	leukemia	acute_myeloid_leukaemia	-1,146771188	0,317660782
1330985	OCI-M1	leukemia	acute_myeloid_leukaemia	-1,108889815	0,329925036
907278	KG-1	leukemia	acute_myeloid_leukaemia	-0,955638274	0,384566607
1330942	ME-1	leukemia	acute_myeloid_leukaemia	-0,397740422	0,671836399
753548	CTV-1	leukemia	acute_myeloid_leukaemia	0,993475633	2,70060449
909744	SW13	kidney	adrenal_gland	-1,691715097	0,184203326
907273	KARPAS-299	lymphoma	anaplastic_large_cell_lymphoma	-1,994687458	0,136056171
909777	U-698-M	leukemia	B_cell_leukemia	-2,792230898	0,061284342
908158	NALM-6	leukemia	B_cell_leukemia	-2,773235761	0,062459573
909696	REH	leukemia	B_cell_leukemia	-1,377373617	0,252240163
910705	BALL-1	leukemia	B_cell_leukemia	-1,022238562	0,359788629
1327774	KARPAS-231	leukemia	B_cell_leukemia	-0,706874336	0,493183316
908132	MHH-CALL-2	leukemia	B_cell_leukemia	-0,442804623	0,642232672
908136	MHH-PREB-1	leukemia	B_cell_leukemia	-0,039971477	0,960816844
908143	MN-60	leukemia	B_cell_leukemia	0,133610173	1,142947181
1330933	KOPN-8	leukemia	B_cell_leukemia	0,139456899	1,149649254
1331037	SU-DHL-6	lymphoma	B_cell_lymphoma	-5,171696642	0,005674932
1327773	KARPAS-1106P	lymphoma	B_cell_lymphoma	-3,626090001	0,026620066

1331033	SU-DHL-10	lymphoma	B_cell_lymphoma	-2,966485572	0,051483929
909773	TUR	lymphoma	B_cell_lymphoma	-2,924337276	0,053700269
1331050	WSU-DLCL2	lymphoma	B_cell_lymphoma	-2,636891234	0,07158346
910934	A4-Fuk	lymphoma	B_cell_lymphoma	-2,428960606	0,088128385
907274	KARPAS-422	lymphoma	B_cell_lymphoma	-2,176013245	0,113493099
1331032	SLVL	lymphoma	B_cell_lymphoma	-1,829990372	0,160415112
910935	A3-KAW	lymphoma	B_cell_lymphoma	-1,770548173	0,170239642
1331048	VAL	lymphoma	B_cell_lymphoma	-1,726326114	0,177936931
906832	DB	lymphoma	B_cell_lymphoma	-1,657533744	0,19060849
910919	BC-1	lymphoma	B_cell_lymphoma	-1,346037357	0,260269575
1331038	SU-DHL-8	lymphoma	B_cell_lymphoma	-1,271970063	0,28027891
1331036	SU-DHL-5	lymphoma	B_cell_lymphoma	-1,10632356	0,330772795
910930	SCC-3	lymphoma	B_cell_lymphoma	-0,859994795	0,423164285
1330995	RC-K8	lymphoma	B_cell_lymphoma	-0,64946062	0,522327434
1331045	TK	lymphoma	B_cell_lymphoma	0,314592415	1,369700928
1327769	JSC-1	lymphoma	B_cell_lymphoma	1,074299756	2,927941909
906853	EGI-1	digestive_system	biliary_tract	-2,036426135	0,130494247
906861	ETK-1	digestive_system	biliary_tract	-1,03915508	0,353753449
909769	TGBC1TKB	digestive_system	biliary_tract	-0,590139742	0,554249827
907069	HuCCT1	digestive_system	biliary_tract	-0,216311742	0,805484159
910695	TGBC24TKB	digestive_system	biliary_tract	-0,182760871	0,832967319
1290730	CAL-29	urogenital_system	Bladder	-2,875572496	0,056383851
910926	BFTC-905	urogenital_system	Bladder	-2,794820036	0,061125874
753584	LB831-BLC	urogenital_system	Bladder	-1,965747217	0,140051199
909749	SW1710	urogenital_system	Bladder	-1,83767879	0,159186503
687459	TCCSUP	urogenital_system	Bladder	-1,480208397	0,227590254
909704	RT-112	urogenital_system	Bladder	-1,095035301	0,334527798
909780	VM-CUB-1	urogenital_system	Bladder	-0,844960533	0,429574312
687452	5637	urogenital_system	Bladder	-0,771800509	0,462180159
724838	UM-UC-3	urogenital_system	Bladder	-0,722548921	0,485513143
906797	647-V	urogenital_system	Bladder	-0,616436323	0,539864918
753552	DSH1	urogenital_system	Bladder	-0,615666356	0,540280756

687455	RT4	urogenital_system	Bladder	-0,552859466	0,575302398
907312	KU-19-19	urogenital_system	Bladder	-0,533872846	0,586329802
753566	J82	urogenital_system	Bladder	-0,39375667	0,674518167
906798	639-V	urogenital_system	Bladder	0,710741162	2,035499334
907066	HT-1376	urogenital_system	Bladder	0,964256009	2,622835564
687457	SW780	urogenital_system	Bladder	3,942467305	51,54562331
1290795	CS1	bone	bone_other	-1,612698776	0,199348889
909717	SJSA-1	bone	bone_other	-0,451309048	0,636794012
908121	MDA-MB-361	breast	breast	-3,309376043	0,036538965
905957	Hs-578-T	breast	breast	-3,098030213	0,045138027
749711	HCC1187	breast	breast	-3,008511591	0,0493651
908122	MDA-MB-453	breast	breast	-2,783083337	0,061847516
905945	T47D	breast	breast	-2,729364083	0,065260777
906851	EFM-19	breast	breast	-2,663747694	0,069686568
908151	MRK-nu-1	breast	breast	-2,588185936	0,075156255
907048	HCC70	breast	breast	-2,541027545	0,078785403
906812	COLO-824	breast	breast	-2,343710274	0,095970899
906844	DU-4475	breast	breast	-2,244921029	0,105935906
946359	BT-474	breast	breast	-2,207988648	0,109921517
749715	HCC2157	breast	breast	-2,143666417	0,117224261
908123	MDA-MB-468	breast	breast	-2,010458423	0,133927265
906801	BT-20	breast	breast	-1,983277044	0,137617519
910852	CAL-85-1	breast	breast	-1,908354061	0,148324319
1290922	HDQ-P1	breast	breast	-1,873720396	0,153551326
946382	CAMA-1	breast	breast	-1,863567074	0,155118324
909256	OCUB-M	breast	breast	-1,851707531	0,156968908
907047	HCC1806	breast	breast	-1,768366862	0,170611393
924106	CAL-148	breast	breast	-1,206738719	0,299171372
749709	HCC1954	breast	breast	-0,971469619	0,37852634
905960	MDA-MB-231	breast	breast	-0,883958127	0,413144393
749710	HCC1143	breast	breast	-0,840758262	0,431383298
905951	BT-549	breast	breast	-0,790107598	0,453795965

924240	MDA-MB-415	breast	breast	-0,75586532	0,469604081
749717	HCC38	breast	breast	-0,752456925	0,471207408
910948	MFM-223	breast	breast	-0,732271242	0,4808157
906862	EVSA-T	breast	breast	-0,658519404	0,517617149
910927	CAL-51	breast	breast	-0,655091331	0,519394624
906826	CAL-120	breast	breast	-0,607714389	0,544594178
910910	UACC-812	breast	breast	-0,421052755	0,656355474
1290798	EFM-192A	breast	breast	-0,206793764	0,813187341
749712	HCC1395	breast	breast	0,065949829	1,068173125
925338	MDA-MB-157	breast	breast	0,143488708	1,154293776
905946	MCF7	breast	breast	0,158391722	1,171625056
910704	AU565	breast	breast	0,195867596	1,216365843
1290905	HCC1428	breast	breast	0,208793542	1,232190577
1303900	HCC1500	breast	breast	0,643630908	1,903379343
907045	HCC1419	breast	breast	0,685565544	1,984894063
749714	HCC1937	breast	breast	0,851244355	2,342560015
949093	BT-483	breast	breast	0,977049605	2,656606629
909907	ZR-75-30	breast	breast	2,234775602	9,344384756
1240172	MDA-MB-436	breast	breast	2,389917159	10,9125899
908159	NAMALWA	lymphoma	Burkitt_lymphoma	-2,927056443	0,053554447
910706	BL-41	lymphoma	Burkitt_lymphoma	-2,704195246	0,06692416
910401	Ramos-2G6-4C10	lymphoma	Burkitt_lymphoma	-2,513007083	0,081024226
906838	DG-75	lymphoma	Burkitt_lymphoma	-1,874788656	0,153387381
906831	Daudi	lymphoma	Burkitt_lymphoma	-1,873800861	0,153538971
906847	EB-3	lymphoma	Burkitt_lymphoma	-1,592020404	0,203514016
910906	ST486	lymphoma	Burkitt_lymphoma	-1,402982537	0,245862575
1303896	GA-10	lymphoma	Burkitt_lymphoma	-1,391073161	0,24880815
906846	EB2	lymphoma	Burkitt_lymphoma	-0,298464792	0,741956404
1330987	P32-ISH	lymphoma	Burkitt_lymphoma	-0,274862569	0,759676519
910703	CA46	lymphoma	Burkitt_lymphoma	0,046476964	1,047573947
909262	Raji	lymphoma	Burkitt_lymphoma	0,390422926	1,47760558
907268	JiyoyeP-2003	lymphoma	Burkitt_lymphoma	0,581679476	1,78904056

924107	CAL-39	urogenital_system	cervix	-3,6449468	0,0261228
906843	DoTc2-4510	urogenital_system	cervix	-2,873685933	0,056490323
906824	Ca-Ski	urogenital_system	cervix	-2,622870587	0,072594176
687514	ME-180	urogenital_system	cervix	-1,359073238	0,256898751
946357	TC-YIK	urogenital_system	cervix	-1,252072978	0,285911494
907068	HT-3	urogenital_system	cervix	-0,716005316	0,488700566
1240212	SISO	urogenital_system	cervix	-0,603719967	0,546773867
930297	SiHa	urogenital_system	cervix	0,332681037	1,394702369
930298	SKG-IIIa	urogenital_system	cervix	0,725130429	2,065000418
1240179	MS751	urogenital_system	cervix	0,751125427	2,119383887
687505	C-33-A	urogenital_system	cervix	0,896310787	2,450545829
724839	SW756	urogenital_system	cervix	3,308827588	27,35303764
907290	H-EMC-SS	bone	chondrosarcoma	-3,06844278	0,046493499
1303912	CHSA8926	bone	chondrosarcoma	-2,4938963	0,082587553
906855	EM-2	leukemia	chronic_myeloid_leukaemia	-2,422110503	0,088734146
1330931	KCL-22	leukemia	chronic_myeloid_leukaemia	-1,98912582	0,136814974
910710	BV-173	leukemia	chronic_myeloid_leukaemia	-1,610153036	0,199857026
910544	RPMI-8866	leukemia	chronic_myeloid_leukaemia	-1,390784971	0,248879864
910951	CML-T1	leukemia	chronic_myeloid_leukaemia	-1,330601261	0,264318289
908126	MEG-01	leukemia	chronic_myeloid_leukaemia	-1,046649796	0,351112078
905940	K-562	leukemia	chronic_myeloid_leukaemia	-0,476385815	0,621023836
907311	KU812	leukemia	chronic_myeloid_leukaemia	0,075296191	1,078203458
907783	LAMA-84	leukemia	chronic_myeloid_leukaemia	0,211652319	1,235718175
1327771	JURL-MK1	leukemia	chronic_myeloid_leukaemia	0,258440478	1,294909072
908129	MFE-280	urogenital_system	endometrium	-3,519509654	0,029613953
909735	SNG-M	urogenital_system	endometrium	-2,482968673	0,083494988
910781	AN3-CA	urogenital_system	endometrium	-2,468148551	0,084741609
924187	KLE	urogenital_system	endometrium	-1,367389599	0,254771147
1240174	MFE-319	urogenital_system	endometrium	-0,55284126	0,575312873
907051	HEC-1	urogenital_system	endometrium	-0,514487917	0,597806643
910691	COLO-684	urogenital_system	endometrium	-0,188195005	0,828453139
930082	RL95-2	urogenital_system	endometrium	-0,030194937	0,970256376

1240127	EN	urogenital_system	endometrium	0,449524853	1,567567184
908130	MFE-296	urogenital_system	endometrium	0,61424954	1,848269027
949164	EW-18	bone	ewings_sarcoma	-3,108461746	0,044669616
949167	EW-22	bone	ewings_sarcoma	-2,574482462	0,076193245
949160	EW-7	bone	ewings_sarcoma	-2,145531782	0,117005799
949156	ES4	bone	ewings_sarcoma	-2,120245545	0,120002159
684059	ES7	bone	ewings_sarcoma	-2,03609875	0,130536976
1240221	TC-71	bone	ewings_sarcoma	-2,013080748	0,133576525
949158	ES1	bone	ewings_sarcoma	-1,97573217	0,138659753
909731	SK-PN-DW	bone	ewings_sarcoma	-1,78918097	0,167096971
949155	ES8	bone	ewings_sarcoma	-1,771933604	0,17000395
949163	EW-1	bone	ewings_sarcoma	-1,770743713	0,170206357
949161	EW-3	bone	ewings_sarcoma	-1,759405955	0,172147097
949168	EW-24	bone	ewings_sarcoma	-1,435831143	0,237917538
684062	EW-11	bone	ewings_sarcoma	-1,218083143	0,295796624
684072	SK-ES-1	bone	ewings_sarcoma	-0,736023577	0,479014899
949165	EW-16	bone	ewings_sarcoma	-0,658527381	0,51761302
949157	ES6	bone	ewings_sarcoma	-0,112649821	0,893463477
949166	EW-13	bone	ewings_sarcoma	2,417454664	11,21727122
909754	SW684	soft_tissue	fibrosarcoma	-0,340127675	0,711679453
907064	HT-1080	soft_tissue	fibrosarcoma	-0,229803223	0,794689964
909905	YH-13	nervous_system	glioma	-3,629187493	0,026537738
907042	H4	nervous_system	glioma	-3,525109601	0,02944858
906871	GI-1	nervous_system	glioma	-3,519866511	0,029603387
687586	T98G	nervous_system	glioma	-3,329261201	0,035819559
906868	GAMG	nervous_system	glioma	-2,540306797	0,078842208
909750	SW1783	nervous_system	glioma	-2,40177311	0,090557243
910694	LN-405	nervous_system	glioma	-2,334132073	0,096894543
907313	KS-1	nervous_system	glioma	-2,134167788	0,118343036
908144	MOG-G-CCM	nervous_system	glioma	-1,955806954	0,141450287
906746	Becker	nervous_system	glioma	-1,953340495	0,141799599
909745	SW1088	nervous_system	glioma	-1,803859556	0,164662137

949094	M059J	nervous_system	glioma	-1,79215436	0,166600865
1240168	LN-18	nervous_system	glioma	-1,730353881	0,177221683
910943	CAS-1	nervous_system	glioma	-1,685773984	0,185300956
908145	MOG-G-UVW	nervous_system	glioma	-1,659792927	0,190178357
946368	D-263MG	nervous_system	glioma	-1,641407609	0,193707186
946370	D-392MG	nervous_system	glioma	-1,475830131	0,22858889
1240170	LNZTA3WT4	nervous_system	glioma	-1,321440245	0,266750839
905984	SF539	nervous_system	glioma	-1,223179836	0,294292874
909729	SK-MG-1	nervous_system	glioma	-1,127369449	0,323884131
906839	DK-MG	nervous_system	glioma	-0,728188679	0,482782673
905983	U251	nervous_system	glioma	-0,569445759	0,565838963
906835	DBTRG-05MG	nervous_system	glioma	-0,444548434	0,641113716
687561	42-MG-BA	nervous_system	glioma	-0,174351787	0,840001344
910933	AM-38	nervous_system	glioma	-0,076352226	0,926489815
687562	8-MG-BA	nervous_system	glioma	0,213262841	1,237709929
1240169	LN-229	nervous_system	glioma	0,25613063	1,291921481
687568	GB-1	nervous_system	glioma	0,471578189	1,60252128
905986	SF268	nervous_system	glioma	0,974232427	2,649133027
907271	KALS-1	nervous_system	glioma	1,024274157	2,785073201
687588	U-118-MG	nervous_system	glioma	1,268805726	3,556602467
687590	U-87-MG	nervous_system	glioma	2,130077981	8,415523037
907279	KINGS-1	nervous_system	glioma	2,405833003	11,08766249
1327775	KARPAS-620	myeloma	haematopoietic_neoplasm_other	-2,990932517	0,050240565
906856	EoL-1-cell	leukemia	haematopoietic_neoplasm_other	-2,077190278	0,125281725
1327766	JJN-3	myeloma	haematopoietic_neoplasm_other	-2,048184861	0,128968788
683665	MC-CAR	myeloma	haematopoietic_neoplasm_other	-2,015398841	0,13326724
908142	MLMA	leukemia	hairy_cell_leukaemia	-2,153720211	0,116051617
907044	HC-1	leukemia	hairy_cell_leukaemia	-0,717015045	0,48820736
753562	HSC-2	aero_dig_tract	head_and_neck	-3,074993996	0,046189906
924111	HO-1-N-1	aero_dig_tract	head_and_neck	-2,918432096	0,054018316
909708	SAS	aero_dig_tract	head_and_neck	-2,597350715	0,074470611
753541	CAL-33	aero_dig_tract	head_and_neck	-2,437309231	0,087395697

910904	SCC-4	aero_dig_tract	head_and_neck	-2,412873025	0,089557623
910911	SCC-15	aero_dig_tract	head_and_neck	-2,396182183	0,09106496
753538	Ca9-22	aero_dig_tract	head_and_neck	-2,386856202	0,091918202
909709	SCC-9	aero_dig_tract	head_and_neck	-2,290922506	0,101173086
1240162	JHU-022	aero_dig_tract	head_and_neck	-2,158073016	0,115547565
907062	HSC-4	aero_dig_tract	head_and_neck	-2,150106038	0,116471807
753561	HO-1-u-1	aero_dig_tract	head_and_neck	-2,089262035	0,123778446
910936	DOK	aero_dig_tract	head_and_neck	-2,080821784	0,124827589
1240121	BICR22	aero_dig_tract	head_and_neck	-2,022481274	0,132326719
1290724	BICR10	aero_dig_tract	head_and_neck	-2,003908098	0,134807412
1240122	BICR78	aero_dig_tract	head_and_neck	-1,972370738	0,139126632
906837	Detroit562	aero_dig_tract	head_and_neck	-1,920960819	0,146466167
1240140	H3118	aero_dig_tract	head_and_neck	-1,751197035	0,173566054
907061	HSC-3	aero_dig_tract	head_and_neck	-1,72610049	0,177977082
753583	LB771-HNC	aero_dig_tract	head_and_neck	-1,519734889	0,218769878
753532	BB49-HNC	aero_dig_tract	head_and_neck	-1,38277684	0,250880929
906794	A253	aero_dig_tract	head_and_neck	-1,284746815	0,276720636
910916	CAL-27	aero_dig_tract	head_and_neck	-1,178348418	0,307786654
1240196	OSC-20	aero_dig_tract	head_and_neck	-1,010549963	0,364018728
1299050	SAT	aero_dig_tract	head_and_neck	-0,988270164	0,372220014
1298529	PCI-30	aero_dig_tract	head_and_neck	-0,235558565	0,790129388
906863	FADU	aero_dig_tract	head_and_neck	-0,040252651	0,960546725
1299059	SKN-3	aero_dig_tract	head_and_neck	1,186851707	3,276748786
1330935	L-1236	lymphoma	Hodgkin_lymphoma	-2,650565891	0,070611243
907323	L-540	lymphoma	Hodgkin_lymphoma	-1,229019407	0,292579338
1331039	SUP-HD1	lymphoma	Hodgkin_lymphoma	-1,085907821	0,337595171
924110	HDLM-2	lymphoma	Hodgkin_lymphoma	-1,025354933	0,358669139
1322218	Hs-445	lymphoma	Hodgkin_lymphoma	0,335083878	1,398057647
907322	L-428	lymphoma	Hodgkin_lymphoma	1,075048697	2,930135586
907050	HD-MY-Z	lymphoma	Hodgkin_lymphoma	1,273640853	3,573840733
753585	LB996-RCC	kidney	kidney	-3,864714767	0,020968903
910952	CAL-54	kidney	kidney	-3,303896846	0,036739719

910920	A704	kidney	kidney	-2,491606679	0,082776864
905948	A498	kidney	kidney	-1,23655811	0,290381963
910698	BFTC-909	kidney	kidney	-0,845825028	0,429203108
1298168	KMRC-1	kidney	kidney	-0,825916287	0,437833627
1298169	KMRC-20	kidney	kidney	-0,269407097	0,763832239
905947	786-0	kidney	kidney	-0,062774203	0,939155508
905980	TK10	kidney	kidney	0,080104353	1,083400118
909250	OS-RC-2	kidney	kidney	0,277836498	1,320270313
909781	VMRC-RCZ	kidney	kidney	0,651920083	1,91922236
909974	RCC10RGB	kidney	kidney	0,664058909	1,94266144
905979	SN12C	kidney	kidney	1,109105192	3,031644441
905950	ACHN	kidney	kidney	1,497541594	4,470684791
924108	CaR-1	large_intestine	large_intestine	-3,977585133	0,018730817
907291	GP5d	large_intestine	large_intestine	-3,686047334	0,025070903
909718	SK-CO-1	large_intestine	large_intestine	-2,780719572	0,061993882
909757	SW948	large_intestine	large_intestine	-2,709765702	0,066552398
1240123	CCK-81	large_intestine	large_intestine	-2,538243143	0,079005079
909748	SW1463	large_intestine	large_intestine	-2,512049588	0,081101843
910700	C2BBe1	large_intestine	large_intestine	-2,329932329	0,097302331
907792	LS-123	large_intestine	large_intestine	-2,217003597	0,108935034
907795	LS-513	large_intestine	large_intestine	-2,01614319	0,13316808
909698	RKO	large_intestine	large_intestine	-1,964742622	0,140191964
1240173	MDST8	large_intestine	large_intestine	-1,837547763	0,159207362
905937	HCT-15	large_intestine	large_intestine	-1,822089894	0,161687488
1290769	CL-11	large_intestine	large_intestine	-1,696421446	0,183338438
905936	HCT-116	large_intestine	large_intestine	-1,658706292	0,190385124
907790	LoVo	large_intestine	large_intestine	-1,61862609	0,198170781
910569	COLO-320-HSR	large_intestine	large_intestine	-1,569071526	0,208238437
907287	HT55	large_intestine	large_intestine	-1,53967425	0,214450947
1659928	SNU-175	large_intestine	large_intestine	-1,526942191	0,217198805
909761	T84	large_intestine	large_intestine	-1,477072709	0,228305026
910554	CW-2	large_intestine	large_intestine	-1,431007223	0,239068006

905939	HT-29	large_intestine	large_intestine		-1,379235094	0,251771061
908482	NCI-H630	large_intestine	large_intestine		-0,933982239	0,392985628
909755	SW837	large_intestine	large_intestine		-0,887504142	0,411681971
998189	LS-180	large_intestine	large_intestine		-0,828896113	0,436530901
908457	NCI-H747	large_intestine	large_intestine		-0,806003161	0,44663965
908458	NCI-H716	large_intestine	large_intestine		-0,607502537	0,544709564
909263	RCM-1	large_intestine	large_intestine		-0,576329658	0,561957161
917486	LS-1034	large_intestine	large_intestine		-0,376639919	0,686163104
905961	COLO-205	large_intestine	large_intestine		-0,314488013	0,730162612
1660035	SNU-61	large_intestine	large_intestine		-0,247803883	0,780513
909751	SW48	large_intestine	large_intestine		-0,228755407	0,795523089
909740	SNU-C2B	large_intestine	large_intestine		-0,104560285	0,900720496
907794	LS-411N	large_intestine	large_intestine		0,108825394	1,114967653
905962	SW620	large_intestine	large_intestine		0,173544698	1,189513854
1660034	SNU-407	large_intestine	large_intestine		0,251962393	1,286547653
909746	SW1116	large_intestine	large_intestine		0,40161697	1,494238885
905971	HCC2998	large_intestine	large_intestine		0,444198295	1,559239644
1240124	CL-40	large_intestine	large_intestine		0,55483537	1,741654233
910905	SNU-C1	large_intestine	large_intestine		0,589125322	1,802411196
909747	SW1417	large_intestine	large_intestine		0,730212901	2,075522441
905989	KM12	large_intestine	large_intestine		1,385671696	3,997510115
1674021	SNU-C5	large_intestine	large_intestine		1,481555911	4,399786034
910689	COLO-678	large_intestine	large_intestine		3,265148714	26,18400463
907300	KY821	leukemia	leukemia		-2,721530185	0,065774031
908156	MV-4-11	leukemia	leukemia		-2,528180054	0,079804127
909703	RS4-11	leukemia	leukemia		-0,245276846	0,78248788
907071	HuH-7	digestive_system	liver		-2,705185683	0,066857908
909737	SNU-423	digestive_system	liver		-1,211036916	0,297888234
907057	HLE	digestive_system	liver		-1,159559908	0,313624174
909738	SNU-449	digestive_system	liver		-0,554065823	0,574608797
1240160	JHH-7	digestive_system	liver		-0,494926167	0,609615915
1298151	JHH-1	digestive_system	liver		-0,26547442	0,766842058

1240159	JHH-6	digestive_system	liver	-0,243722803	0,783704845
1240217	SNU-398	digestive_system	liver	-0,199222154	0,819367847
1298146	huH-1	digestive_system	liver	0,10615749	1,111996991
909736	SNU-387	digestive_system	liver	0,121424894	1,12910456
909719	SK-HEP-1	digestive_system	liver	0,391148153	1,478677568
1240216	SNU-182	digestive_system	liver	0,587215877	1,798972875
910850	C3A	digestive_system	liver	1,880391287	6,556069665
1240145	HCC-44	lung_NSCLC	lung_NSCLC_adenocarcinoma	-3,209261621	0,040386423
687799	NCI-H1648	lung_NSCLC	lung_NSCLC_adenocarcinoma	-2,868906667	0,056760951
908474	NCI-H1703	lung_NSCLC	lung_NSCLC_adenocarcinoma	-2,613543782	0,073274415
687798	NCI-H1623	lung_NSCLC	lung_NSCLC_adenocarcinoma	-2,236381624	0,106844409
905949	A549	lung_NSCLC	lung_NSCLC_adenocarcinoma	-2,228161546	0,107726298
724873	NCI-H2009	lung_NSCLC	lung_NSCLC_adenocarcinoma	-2,195038758	0,111354246
724866	NCI-H1355	lung_NSCLC	lung_NSCLC_adenocarcinoma	-2,150745291	0,116397376
1503370	EMC-BAC-2	lung_NSCLC	lung_NSCLC_adenocarcinoma	-1,913958769	0,147495329
1240146	HCC-827	lung_NSCLC	lung_NSCLC_adenocarcinoma	-1,693350598	0,183902308
908465	NCI-H358	lung_NSCLC	lung_NSCLC_adenocarcinoma	-1,6277154	0,196377707
905942	NCI-H23	lung_NSCLC	lung_NSCLC_adenocarcinoma	-1,605470884	0,200794982
753600	NCI-H1563	lung_NSCLC	lung_NSCLC_adenocarcinoma	-1,567585876	0,208548036
908475	NCI-H1755	lung_NSCLC	lung_NSCLC_adenocarcinoma	-1,506688061	0,221642831
1240187	NCI-H2023	lung_NSCLC	lung_NSCLC_adenocarcinoma	-1,503753505	0,22229421
724868	NCI-H1792	lung_NSCLC	lung_NSCLC_adenocarcinoma	-1,480842657	0,227445949
1298347	NCI-H1435	lung_NSCLC	lung_NSCLC_adenocarcinoma	-1,392075894	0,248558787
687812	NCI-H2085	lung_NSCLC	lung_NSCLC_adenocarcinoma	-1,298700618	0,272886146
1240185	NCI-H1944	lung_NSCLC	lung_NSCLC_adenocarcinoma	-1,263038025	0,282793586
722046	NCI-H2122	lung_NSCLC	lung_NSCLC_adenocarcinoma	-1,139295499	0,320044414
753608	PC-14	lung_NSCLC	lung_NSCLC_adenocarcinoma	-1,052273277	0,349143147
724859	Calu-6	lung_NSCLC	lung_NSCLC_adenocarcinoma	-1,038356714	0,354035987
905944	NCI-H522	lung_NSCLC	lung_NSCLC_adenocarcinoma	-0,945072569	0,388651366
724834	NCI-H2087	lung_NSCLC	lung_NSCLC_adenocarcinoma	-0,94295733	0,389474326
753592	LXF-289	lung_NSCLC	lung_NSCLC_adenocarcinoma	-0,834661383	0,434021424
1240202	PC-3_[JPC-3]	lung_NSCLC	lung_NSCLC_adenocarcinoma	-0,779196815	0,458774344

908459	NCI-H596	lung_NSCLC	lung_NSCLC_adenocarcinoma	-0,757363848	0,468900893
908473	NCI-H1666	lung_NSCLC	lung_NSCLC_adenocarcinoma	-0,735528875	0,479251928
1290908	HCC-78	lung_NSCLC	lung_NSCLC_adenocarcinoma	-0,692616002	0,50026566
908476	NCI-H1993	lung_NSCLC	lung_NSCLC_adenocarcinoma	-0,644547134	0,524900198
908472	NCI-H1573	lung_NSCLC	lung_NSCLC_adenocarcinoma	-0,569346804	0,565894958
910900	NCI-H1651	lung_NSCLC	lung_NSCLC_adenocarcinoma	-0,088682683	0,915135916
905967	NCI-H322M	lung_NSCLC	lung_NSCLC_adenocarcinoma	-0,04094913	0,959877958
687802	NCI-H1693	lung_NSCLC	lung_NSCLC_adenocarcinoma	0,014706323	1,014814993
905972	HOP-62	lung_NSCLC	lung_NSCLC_adenocarcinoma	0,118867965	1,126221208
1240190	NCI-H3122	lung_NSCLC	lung_NSCLC_adenocarcinoma	0,15501863	1,167679715
1298350	NCI-H1781	lung_NSCLC	lung_NSCLC_adenocarcinoma	0,209207693	1,232700995
908460	NCI-H441	lung_NSCLC	lung_NSCLC_adenocarcinoma	0,290621522	1,337258365
1298537	RERF-LC-KJ	lung_NSCLC	lung_NSCLC_adenocarcinoma	0,601171844	1,824255291
687819	NCI-H2342	lung_NSCLC	lung_NSCLC_adenocarcinoma	0,665414312	1,945296314
909721	SK-LU-1	lung_NSCLC	lung_NSCLC_adenocarcinoma	0,842356429	2,321831767
924244	NCI-H1975	lung_NSCLC	lung_NSCLC_adenocarcinoma	1,104357681	3,017285786
908463	NCI-H1793	lung_NSCLC	lung_NSCLC_adenocarcinoma	1,313421922	3,718877673
687816	NCI-H2228	lung_NSCLC	lung_NSCLC_adenocarcinoma	1,324826362	3,761532153
687807	NCI-H1838	lung_NSCLC	lung_NSCLC_adenocarcinoma	1,440752054	4,223871202
906805	COR-L105	lung_NSCLC	lung_NSCLC_adenocarcinoma	1,501263854	4,487356852
724878	SW1573	lung_NSCLC	lung_NSCLC_adenocarcinoma	2,109899402	8,24741157
722066	NCI-H650	lung_NSCLC	lung_NSCLC_adenocarcinoma	2,920168043	18,54440346
687777	Calu-3	lung_NSCLC	lung_NSCLC_adenocarcinoma	3,000397701	20,09352655
687800	NCI-H1650	lung_NSCLC	lung_NSCLC_adenocarcinoma	3,484052476	32,59153122
687820	NCI-H2347	lung_NSCLC	lung_NSCLC_adenocarcinoma	5,126727813	168,4649663
687821	NCI-H2405	lung_NSCLC	lung_NSCLC_adenocarcinoma	5,278591408	196,093465
687600	NCI-H720	lung_NSCLC	lung_NSCLC_carcinoid	-2,013019415	0,133584717
909779	UMC-11	lung_NSCLC	lung_NSCLC_carcinoid	-1,366168694	0,255082388
724855	NCI-H727	lung_NSCLC	lung_NSCLC_carcinoid	-0,47772792	0,620190916
753607	NCI-H835	lung_NSCLC	lung_NSCLC_carcinoid	-0,352122764	0,70319379
908471	NCI-H1581	lung_NSCLC	lung_NSCLC_large_cell	-1,395392099	0,24773588
687780	COR-L23	lung_NSCLC	lung_NSCLC_large_cell	-1,257342988	0,284408701

925341	NCI-H810	lung_NSCLC	lung_NSCLC_large_cell	-1,233255416	0,291342591
907796	LU-99A	lung_NSCLC	lung_NSCLC_large_cell	-0,876444233	0,416260408
724831	NCI-H1299	lung_NSCLC	lung_NSCLC_large_cell	-0,837945365	0,432598443
724863	LU-65	lung_NSCLC	lung_NSCLC_large_cell	-0,809962931	0,444874557
753586	LCLC-103H	lung_NSCLC	lung_NSCLC_large_cell	-0,701702939	0,495740369
946361	LCLC-97TM1	lung_NSCLC	lung_NSCLC_large_cell	-0,357620139	0,699338676
908467	NCI-H1155	lung_NSCLC	lung_NSCLC_large_cell	0,004972412	1,004984795
910779	IA-LM	lung_NSCLC	lung_NSCLC_large_cell	0,894784669	2,446808859
1240184	NCI-H1915	lung_NSCLC	lung_NSCLC_large_cell	1,142945546	3,135991983
905973	HOP-92	lung_NSCLC	lung_NSCLC_large_cell	2,011959615	7,477956915
687829	NCI-H661	lung_NSCLC	lung_NSCLC_large_cell	3,31881852	27,62768971
687815	NCI-H2170	lung_NSCLC	lung_NSCLC_squamous_cell_carcinoma	-2,065507813	0,126753907
1240142	HARA	lung_NSCLC	lung_NSCLC_squamous_cell_carcinoma	-1,526442885	0,217307281
753554	EBC-1	lung_NSCLC	lung_NSCLC_squamous_cell_carcinoma	-1,492422173	0,224827425
1298538	RERF-LC-Sq1	lung_NSCLC	lung_NSCLC_squamous_cell_carcinoma	-1,331105018	0,264185171
687787	LK-2	lung_NSCLC	lung_NSCLC_squamous_cell_carcinoma	-1,050433112	0,34978622
909728	SK-MES-1	lung_NSCLC	lung_NSCLC_squamous_cell_carcinoma	-0,861109525	0,422692834
753569	KNS-62	lung_NSCLC	lung_NSCLC_squamous_cell_carcinoma	-0,85394428	0,425732408
908443	NCI-H520	lung_NSCLC	lung_NSCLC_squamous_cell_carcinoma	-0,320501999	0,725784602
1240143	HCC-15	lung_NSCLC	lung_NSCLC_squamous_cell_carcinoma	-0,305851102	0,736496274
724879	SW900	lung_NSCLC	lung_NSCLC_squamous_cell_carcinoma	-0,250985718	0,778033483
753556	EPLC-272H	lung_NSCLC	lung_NSCLC_squamous_cell_carcinoma	0,120637388	1,128215734
1298223	LC-1-sq	lung_NSCLC	lung_NSCLC_squamous_cell_carcinoma	0,137375913	1,147259337
1298226	LOU-NH91	lung_NSCLC	lung_NSCLC_squamous_cell_carcinoma	0,894567208	2,446276831
905941	NCI-H226	lung_NSCLC	lung_NSCLC_squamous_cell_carcinoma	1,318420581	3,737513613
1240183	NCI-H1869	lung_NSCLC	lung_NSCLC_squamous_cell_carcinoma	1,625332222	5,08010648
1240193	NCI-H847	lung_SCLC	lung_small_cell_carcinoma	-3,069722813	0,046434024
687980	COR-L311	lung_SCLC	lung_small_cell_carcinoma	-3,043448165	0,047670231
688001	NCI-H1417	lung_SCLC	lung_small_cell_carcinoma	-2,9630682	0,05166017
1330972	NCI-H1876	lung_SCLC	lung_small_cell_carcinoma	-2,927327194	0,053539949
688025	NCI-H526	lung_SCLC	lung_small_cell_carcinoma	-2,685643727	0,068177292
753610	SBC-3	lung_SCLC	lung_small_cell_carcinoma	-2,208804754	0,109831846

908483	NCI-H524	lung_SCLC	lung_small_cell_carcinoma	-2,045044746	0,129374401
910937	COR-L279	lung_SCLC	lung_small_cell_carcinoma	-1,907982646	0,148379419
688027	NCI-H69	lung_SCLC	lung_small_cell_carcinoma	-1,847947168	0,157560279
753594	MS-1	lung_SCLC	lung_small_cell_carcinoma	-1,716752107	0,179648681
688031	NCI-H82	lung_SCLC	lung_small_cell_carcinoma	-1,676751684	0,186980362
713899	LU-135	lung_SCLC	lung_small_cell_carcinoma	-1,660315094	0,190079078
1330964	NCI-H1341	lung_SCLC	lung_small_cell_carcinoma	-1,590927807	0,203736496
1240192	NCI-H841	lung_SCLC	lung_small_cell_carcinoma	-1,404634644	0,245456719
753547	CPC-N	lung_SCLC	lung_small_cell_carcinoma	-1,210768672	0,297968152
688014	NCI-H2141	lung_SCLC	lung_small_cell_carcinoma	-1,158780138	0,313868824
1330973	NCI-H2066	lung_SCLC	lung_small_cell_carcinoma	-1,145664849	0,318012417
687995	NCI-H1048	lung_SCLC	lung_small_cell_carcinoma	-1,137079812	0,320754318
910692	COLO-668	lung_SCLC	lung_small_cell_carcinoma	-1,11644564	0,327441574
713878	LU-139	lung_SCLC	lung_small_cell_carcinoma	-0,969964059	0,379096663
688023	NCI-H446	lung_SCLC	lung_small_cell_carcinoma	-0,962110126	0,382085786
688006	NCI-H1694	lung_SCLC	lung_small_cell_carcinoma	-0,810598876	0,444591731
687983	DMS-114	lung_SCLC	lung_small_cell_carcinoma	-0,768888938	0,46352779
688013	NCI-H209	lung_SCLC	lung_small_cell_carcinoma	-0,468704812	0,625812288
688026	NCI-H64	lung_SCLC	lung_small_cell_carcinoma	-0,460014914	0,631274231
907295	DMS-53	lung_SCLC	lung_small_cell_carcinoma	-0,323329452	0,723735379
1240189	NCI-H211	lung_SCLC	lung_small_cell_carcinoma	-0,283430588	0,753195401
687997	NCI-H1092	lung_SCLC	lung_small_cell_carcinoma	-0,251072901	0,777965655
1240186	NCI-H196	lung_SCLC	lung_small_cell_carcinoma	-0,211800835	0,809125831
1298349	NCI-H1688	lung_SCLC	lung_small_cell_carcinoma	-0,065017128	0,937051413
687985	DMS-273	lung_SCLC	lung_small_cell_carcinoma	0,129691328	1,138476913
724872	SHP-77	lung_SCLC	lung_small_cell_carcinoma	0,430279622	1,537687435
1297439	COR-L95	lung_SCLC	lung_small_cell_carcinoma	0,437177533	1,548330932
713880	SBC-5	lung_SCLC	lung_small_cell_carcinoma	0,522044633	1,685470297
688010	NCI-H1963	lung_SCLC	lung_small_cell_carcinoma	0,535479444	1,708267064
1299062	SW1271	lung_SCLC	lung_small_cell_carcinoma	0,752190269	2,121641898
753589	LU-165	lung_SCLC	lung_small_cell_carcinoma	1,265655594	3,545416328
906808	COR-L88	lung_SCLC	lung_small_cell_carcinoma	1,814517783	6,13811556

949153	HAL-01	leukemia	lymphoblastic_leukemia	-3,501360535	0,030156327
910944	ALL-PO	leukemia	lymphoblastic_leukemia	-3,026554066	0,048482418
1247871	SUP-B15	leukemia	lymphoblastic_leukemia	-2,915989501	0,054150423
905958	MOLT-4	leukemia	lymphoblastic_leukemia	-2,471281645	0,084476521
906877	GR-ST	leukemia	lymphoblastic_leukemia	-2,205223577	0,110225879
907272	KARPAS-45	leukemia	lymphoblastic_leukemia	-2,173512334	0,11377729
907787	LC4-1	leukemia	lymphoblastic_leukemia	-2,118498801	0,120211955
906763	BE-13	leukemia	lymphoblastic_leukemia	-1,917681728	0,146947231
907277	KE-37	leukemia	lymphoblastic_leukemia	-1,890821123	0,150947811
909252	P30-OHK	leukemia	lymphoblastic_leukemia	-1,600104895	0,201875341
906800	697	leukemia	lymphoblastic_leukemia	-1,385082802	0,250303073
905952	CCRF-CEM	leukemia	lymphoblastic_leukemia	-1,215720778	0,296496229
908147	MOLT-16	leukemia	lymphoblastic_T_cell_leukaemia	-3,347339267	0,035177828
910687	ATN-1	leukemia	lymphoblastic_T_cell_leukaemia	-2,707290664	0,066717322
909702	RPMI-8402	leukemia	lymphoblastic_T_cell_leukaemia	-2,64731523	0,07084115
909743	SUP-T1	leukemia	lymphoblastic_T_cell_leukaemia	-1,877403972	0,152986749
907789	LOUCY	leukemia	lymphoblastic_T_cell_leukaemia	-1,785270557	0,167751668
909251	P12-ICHIKAWA	leukemia	lymphoblastic_T_cell_leukaemia	-1,134110852	0,32170804
909260	PF-382	leukemia	lymphoblastic_T_cell_leukaemia	-0,88756733	0,411655959
908146	MOLT-13	leukemia	lymphoblastic_T_cell_leukaemia	-0,162999851	0,849591315
909785	WSU-NHL	lymphoma	lymphoid_neoplasm_other	-5,336176433	0,004814243
946358	YT	lymphoma	lymphoid_neoplasm_other	-2,465932448	0,084929613
907270	JVM-3	lymphoma	lymphoid_neoplasm_other	-2,416677376	0,089217562
907043	H9	lymphoma	lymphoid_neoplasm_other	-1,844497982	0,158104672
1331049	WIL2-NS	lymphoma	lymphoid_neoplasm_other	-1,344265713	0,260731089
906836	DEL	lymphoma	lymphoid_neoplasm_other	-1,189058597	0,304507794
1297447	EJM	myeloma	lymphoid_neoplasm_other	-0,625566058	0,534958525
906834	D-283MED	nervous_system	medulloblastoma	-3,33029133	0,035782679
906833	Daoy	nervous_system	medulloblastoma	-0,503847237	0,604201675
909248	ONS-76	nervous_system	medulloblastoma	0,334247733	1,396889156
905974	LOXIMVI	skin	melanoma	-3,048129574	0,047447589
1299078	WM1552C	skin	melanoma	-2,509320384	0,081323489

910903	RPMI-7951	skin	melanoma	-2,445458231	0,086686403
907172	IST-MEL1	skin	melanoma	-2,406223803	0,090155096
910853	CHL-1	skin	melanoma	-1,990523574	0,136623874
910921	A101D	skin	melanoma	-1,812019941	0,163323899
906792	A2058	skin	melanoma	-1,740624105	0,175410892
908128	Mewo	skin	melanoma	-1,251720851	0,286012189
907169	IGR-1	skin	melanoma	-1,120666165	0,326062511
906814	COLO-792	skin	melanoma	-1,112162408	0,32884709
909725	SK-MEL-24	skin	melanoma	-0,819685727	0,440570092
1240153	IGR-37	skin	melanoma	-0,746038946	0,474241333
1299081	WM793B	skin	melanoma	-0,685933051	0,503620107
906793	A375	skin	melanoma	-0,475439311	0,621611916
909713	SH-4	skin	melanoma	-0,376172413	0,686483964
910932	GAK	skin	melanoma	-0,358798422	0,698515142
909784	WM-115	skin	melanoma	-0,34713143	0,706712439
907067	HT-144	skin	melanoma	-0,197701309	0,820614927
905955	SK-MEL-2	skin	melanoma	-0,132013222	0,876329408
905976	UACC-62	skin	melanoma	-0,063309611	0,938652811
909723	SK-MEL-1	skin	melanoma	0,124699895	1,132808441
906865	G-361	skin	melanoma	0,265473516	1,304048317
907058	HMV-II	skin	melanoma	0,306986292	1,359322334
908125	MEL-JUSO	skin	melanoma	0,584249508	1,793644365
905975	M14	skin	melanoma	0,666078183	1,946588169
1299080	WM35	skin	melanoma	0,672513999	1,959156452
906830	C32	skin	melanoma	0,877090589	2,403895602
905977	UACC-257	skin	melanoma	1,475873001	4,374853358
1240226	WM278	skin	melanoma	1,919026223	6,81431961
906818	COLO-679	skin	melanoma	2,977835121	19,64524101
687448	COLO-829	skin	melanoma	3,410590699	30,28312719
908150	MPP-89	lung	mesothelioma	-2,199933976	0,110810474
1290813	H2795	lung	mesothelioma	-0,946877277	0,387950596
908462	NCI-H2452	lung	mesothelioma	-0,884945916	0,412736495

1290810	H2461	lung	mesothelioma	-0,769616964	0,463190453
1240141	H513	lung	mesothelioma	-0,605479457	0,54581267
1240134	H2731	lung	mesothelioma	-0,240206731	0,786465258
908470	NCI-H28	lung	mesothelioma	-0,088616956	0,915196067
1290814	H2818	lung	mesothelioma	1,135953722	3,114142153
688058	NCI-H2052	lung	mesothelioma	1,404331919	4,072804869
1290812	H2722	lung	mesothelioma	2,034068424	7,64512679
1240132	H2595	lung	mesothelioma	2,194218895	8,972989472
907173	IST-MES1	lung	mesothelioma	2,434385932	11,40881078
1240131	H2591	lung	mesothelioma	5,783150676	324,7808594
724825	NCI-H929	myeloma	myeloma	-3,328639343	0,03584184
905964	RPMI-8226	myeloma	myeloma	-2,942884381	0,052713464
907281	KMS-12-BM	myeloma	myeloma	-2,790627688	0,061382673
1330950	MOLP-8	myeloma	myeloma	-2,75398209	0,063673801
753563	IM-9	myeloma	myeloma	-2,217769162	0,108851669
909249	OPM-2	myeloma	myeloma	-1,783460553	0,168055575
1659818	MM1S	myeloma	myeloma	-1,606817363	0,200524797
924239	L-363	myeloma	myeloma	-1,455088915	0,233379612
906765	ARH-77	myeloma	myeloma	-1,049133504	0,3502411
753615	U-266	myeloma	myeloma	-0,933037373	0,393357122
753612	SK-MM-2	myeloma	myeloma	-0,481618165	0,617782908
910780	TGW	neuroblastoma	neuroblastoma	-4,305238578	0,013497665
949178	NB14	neuroblastoma	neuroblastoma	-4,149448648	0,015773111
906820	CHP-212	neuroblastoma	neuroblastoma	-3,586922465	0,027683396
949172	NB12	neuroblastoma	neuroblastoma	-3,509035077	0,029925777
906875	GOTO	neuroblastoma	neuroblastoma	-3,386447576	0,033828637
908447	NH-12	neuroblastoma	neuroblastoma	-3,231114533	0,039513435
1240181	NB(TU)1-10	neuroblastoma	neuroblastoma	-3,117854837	0,044251994
717431	SK-N-SH	neuroblastoma	neuroblastoma	-3,085670427	0,045699386
949174	NB7	neuroblastoma	neuroblastoma	-2,94783977	0,052452894
753616	BE2-M17	neuroblastoma	neuroblastoma	-2,607431434	0,073723665
949177	NB13	neuroblastoma	neuroblastoma	-2,399260957	0,090785023

753618	KELLY	neuroblastoma	neuroblastoma	-2,207741479	0,10994869
688086	SK-N-DZ	neuroblastoma	neuroblastoma	-2,035240077	0,130649113
688087	SK-N-FI	neuroblastoma	neuroblastoma	-1,786817983	0,167492286
907170	IMR-5	neuroblastoma	neuroblastoma	-0,744715549	0,474869358
724828		neuroblastoma	neuroblastoma	-0,131451328	0,876821951
1299064	T-T	aero_dig_tract	oesophagus	-3,046929217	0,047504577
907319	KYSE-270	aero_dig_tract	oesophagus	-3,046506992	0,047524639
1503368	KYAE-1	aero_dig_tract	oesophagus	-2,819936675	0,059609717
753555	EC-GI-10	aero_dig_tract	oesophagus	-2,321443702	0,09813181
1503361	FLO-1	aero_dig_tract	oesophagus	-2,232756905	0,107232393
753614	TE-15	aero_dig_tract	oesophagus	-2,120804915	0,119935052
753623	TE-8	aero_dig_tract	oesophagus	-1,888868222	0,151242885
1503365	SK-GT-4	aero_dig_tract	oesophagus	-1,823622903	0,161439809
1503371	TE-4	aero_dig_tract	oesophagus	-1,650564341	0,191941558
1298222	KYSE-50	aero_dig_tract	oesophagus	-1,576636442	0,206669074
735784	TE-5	aero_dig_tract	oesophagus	-1,538555255	0,214691051
946353	TE-9	aero_dig_tract	oesophagus	-1,469437475	0,23005486
907320	KYSE-450	aero_dig_tract	oesophagus	-1,388974064	0,249330971
753621	TE-1	aero_dig_tract	oesophagus	-1,180311525	0,307183029
753575	KYSE-520	aero_dig_tract	oesophagus	-0,935588407	0,392354933
910549	OE33	aero_dig_tract	oesophagus	-0,85238435	0,426397039
1240167	KYSE-220	aero_dig_tract	oesophagus	-0,72213815	0,485712618
1503363	OACM5-1	aero_dig_tract	oesophagus	-0,681593015	0,505810586
910079	OE19	aero_dig_tract	oesophagus	-0,676309965	0,50848988
907317	KYSE-150	aero_dig_tract	oesophagus	-0,520615082	0,594154982
753573	KYSE-140	aero_dig_tract	oesophagus	-0,393575912	0,674640102
907318	KYSE-180	aero_dig_tract	oesophagus	-0,311412544	0,732411661
1503366	ESO26	aero_dig_tract	oesophagus	-0,308185811	0,734778775
753576	KYSE-70	aero_dig_tract	oesophagus	0,24874752	1,282418207
1503367	ESO51	aero_dig_tract	oesophagus	0,293430808	1,341020388
1298359	OE21	aero_dig_tract	oesophagus	0,314409796	1,369450817
946355	TE-6	aero_dig_tract	oesophagus	0,341879413	1,40759055

906817	COLO-680N	aero_dig_tract	oesophagus	0,979415422	2,662899115
753574	KYSE-410	aero_dig_tract	oesophagus	1,419124739	4,133500964
909707	Saos-2	bone	osteosarcoma	-2,405492491	0,090221052
909977	HuO-3N1	bone	osteosarcoma	-2,289171261	0,10135042
925345	NOS-1	bone	osteosarcoma	-2,084121767	0,124416339
910849	NY	bone	osteosarcoma	-1,860498567	0,155595036
906827	CAL-72	bone	osteosarcoma	-1,542405402	0,213866048
907072	HuO9	bone	osteosarcoma	-1,27975121	0,278106482
907060	HOS	bone	osteosarcoma	-1,202690831	0,300384839
909776	U-2-OS	bone	osteosarcoma	0,4995679	1,648009012
909255	PA-1	urogenital_system	ovary	-3,287549132	0,037345266
905968	IGROV-1	urogenital_system	ovary	-2,672227122	0,069098164
1480372	PEO1	urogenital_system	ovary	-2,379608445	0,092586823
1479987	DOV13	urogenital_system	ovary	-2,203529841	0,11041273
1240129	FU-OV-1	urogenital_system	ovary	-2,161872031	0,115109431
1240222	TOV-21G	urogenital_system	ovary	-2,155286666	0,11586997
905933	OVCAR-3	urogenital_system	ovary	-1,985657705	0,137290288
1298365	OVTOKO	urogenital_system	ovary	-1,968740651	0,139632592
1480362	OV-56	urogenital_system	ovary	-1,904172694	0,148945815
909975	KURAMOCHI	urogenital_system	ovary	-1,831428981	0,160184504
910548	OAW-42	urogenital_system	ovary	-1,701146993	0,182474107
949090	Caov-4	urogenital_system	ovary	-1,6702779	0,188194759
1240200	OVMIU	urogenital_system	ovary	-1,611412764	0,199605419
946360	OAW-28	urogenital_system	ovary	-1,609058399	0,200075917
906804	A2780	urogenital_system	ovary	-1,443660234	0,236062133
905969	OVCAR-5	urogenital_system	ovary	-1,419146489	0,241920411
909699	RMG-I	urogenital_system	ovary	-1,350977936	0,258986864
1240197	OV-90	urogenital_system	ovary	-1,325464733	0,26567946
1299070	TOV-112D	urogenital_system	ovary	-1,209126476	0,298457876
909257	OC-314	urogenital_system	ovary	-1,061552172	0,345918468
924186	KGN	urogenital_system	ovary	-0,79055982	0,453590795
1298539	RKN	urogenital_system	ovary	-0,736075942	0,478989816

905991	OVCAR-8	urogenital_system	ovary	-0,405901809	0,666375596
909774	TYK-nu	urogenital_system	ovary	0,015500577	1,015621334
1240199	OVKATE	urogenital_system	ovary	0,281264301	1,324803705
1240198	OVISE	urogenital_system	ovary	0,34068541	1,405910886
1480364	OVCA420	urogenital_system	ovary	0,423035209	1,526588045
909753	SW626	urogenital_system	ovary	0,468380204	1,597404627
1479988	Hey	urogenital_system	ovary	0,499403556	1,647738194
905990	OVCAR-4	urogenital_system	ovary	0,678719663	1,971352121
905959	SK-OV-3	urogenital_system	ovary	0,985288053	2,678583347
1240128	ES-2	urogenital_system	ovary	1,638738372	5,148669707
1290797	DAN-G	pancreas	pancreas	-1,74731325	0,174241458
925346	PANC-03-27	pancreas	pancreas	-1,706546515	0,18149149
753624	CAPAN-1	pancreas	pancreas	-1,553420878	0,21152314
724869	HPAF-II	pancreas	pancreas	-1,525686423	0,217471728
907286	HuP-T4	pancreas	pancreas	-1,516324601	0,219517219
753572	KP-4	pancreas	pancreas	-1,514321017	0,219957482
1298136	HPAC	pancreas	pancreas	-1,398085696	0,247069478
1298216	KP-1N	pancreas	pancreas	-1,354313942	0,258124322
906693	BxPC-3	pancreas	pancreas	-1,021521771	0,360046615
1298526	PA-TU-8902	pancreas	pancreas	-0,876707561	0,41615081
907285	HuP-T3	pancreas	pancreas	-0,814143669	0,443018535
910915	Capan-2	pancreas	pancreas	-0,690050703	0,501550638
925348	PANC-10-05	pancreas	pancreas	-0,588439469	0,555193005
906821	CFPAC-1	pancreas	pancreas	-0,473991902	0,622512294
1298475	PANC-02-03	pancreas	pancreas	-0,337128293	0,713817256
1298533	PL4	pancreas	pancreas	-0,198421367	0,820024249
1240208	PL18	pancreas	pancreas	-0,193277986	0,824252812
909904	YAPC	pancreas	pancreas	0,359856821	1,433124207
1298476	PANC-04-03	pancreas	pancreas	0,368434642	1,445470165
910546	PSN1	pancreas	pancreas	0,468234984	1,597172669
925347	PANC-08-13	pancreas	pancreas	0,680221565	1,974315123
1240201	PA-TU-8988T	pancreas	pancreas	0,873749455	2,395877267

1240218	SU8686	pancreas	pancreas	1,033819199	2,811784117
1298141	Hs-766T	pancreas	pancreas	1,058669563	2,882533408
910907	SW1990	pancreas	pancreas	1,210045878	3,353638507
1240219	SUIT-2	pancreas	pancreas	2,000518683	7,392889671
724870	MIA-PaCa-2	pancreas	pancreas	3,094697874	22,08056642
1298219	KP-3	pancreas	pancreas	4,317944356	75,03422599
910702	AsPC-1	pancreas	pancreas	5,253580292	191,2497742
1299075	VCaP	urogenital_system	prostate	-1,808352622	0,163923959
907788	LNCaP-Clone-FGC	urogenital_system	prostate	-1,23842222	0,289841163
924100	22RV1	urogenital_system	prostate	-0,980889549	0,37497739
905934	PC-3	urogenital_system	prostate	-0,15330835	0,857865158
905935	DU-145	urogenital_system	prostate	0,286573167	1,331855612
684052	A673	soft_tissue	rhabdomyosarcoma	-2,133396086	0,118434397
909264	RD	soft_tissue	rhabdomyosarcoma	-1,734968546	0,176405749
1240166	KYM-1	soft_tissue	rhabdomyosarcoma	-1,555167663	0,211153977
1240210	RH-41	soft_tissue	rhabdomyosarcoma	-0,929679959	0,394680004
910784	A204	soft_tissue	rhabdomyosarcoma	-0,902871681	0,405403796
971773	RH-1	soft_tissue	rhabdomyosarcoma	-0,780365881	0,45823832
909716	SJRH30	soft_tissue	rhabdomyosarcoma	-0,477640342	0,620245233
909756	SW872	soft_tissue	soft_tissue_other	-2,299492346	0,100309753
909759	SW982	soft_tissue	soft_tissue_other	-1,402975589	0,245864283
909732	SK-UT-1	soft_tissue	soft_tissue_other	-1,000720498	0,36761448
906999	GCT	soft_tissue	soft_tissue_other	-0,945190268	0,388605625
909720	SK-LMS-1	soft_tissue	soft_tissue_other	-0,73284097	0,480541844
688121	VA-ES-BJ	soft_tissue	soft_tissue_other	-0,224009864	0,79930725
925343	MFH-ino	soft_tissue	soft_tissue_other	1,464319569	4,324599647
906790	AGS	digestive_system	stomach	-3,48241553	0,030733085
906869	GCIY	digestive_system	stomach	-2,964210201	0,051601208
907055	HGC-27	digestive_system	stomach	-2,909045917	0,054527729
1322224	HSC-39	digestive_system	stomach	-2,895347823	0,055279794
1290806	FU97	digestive_system	stomach	-2,862597932	0,057120173
910924	23132-87	digestive_system	stomach	-2,278388294	0,102449192

908446	NCI-SNU-16	digestive_system	stomach	-2,20081302	0,11071311
908461	NCI-N87	digestive_system	stomach	-1,864608267	0,1549569
908445	NCI-SNU-5	digestive_system	stomach	-1,845111288	0,158007736
1240209	RERF-GC-1B	digestive_system	stomach	-1,809299886	0,163768753
909770	TGBC11TKB	digestive_system	stomach	-1,585039615	0,204939674
908444	NCI-SNU-1	digestive_system	stomach	-1,541570033	0,21404478
909697	RF-48	digestive_system	stomach	-1,327259905	0,265202948
908138	MKN1	digestive_system	stomach	-1,185625214	0,305555082
1503364	SK-GT-2	digestive_system	stomach	-0,91765655	0,399454046
908455	NUGC-3	digestive_system	stomach	-0,8571901	0,424352798
906848	ECC10	digestive_system	stomach	-0,741336643	0,476476611
1240155	IM-95	digestive_system	stomach	-0,739897961	0,477162602
925340	MKN45	digestive_system	stomach	-0,665430701	0,514052077
1240151	Hs746T	digestive_system	stomach	0,396978259	1,487323594
908139	MKN28	digestive_system	stomach	0,985834819	2,680048306
1298358	OCUM-1	digestive_system	stomach	2,233106113	9,328797423
907276	KATOIII	digestive_system	stomach	4,11087559	61,00010528
1298357	NUGC-4	digestive_system	stomach	6,004783257	405,3631196
998184	Jurkat	leukemia	T_cell_leukemia	-2,416399912	0,08924232
1297446	DND-41	leukemia	T_cell_leukemia	-1,616338806	0,198624573
907056	HH	leukemia	T_cell_leukemia	-1,027235024	0,357995442
1295740	ALL-SIL	leukemia	T_cell_leukemia	-0,955787571	0,384509197
908454	NTERA-S-cl-D1	urogenital_system	testis	-3,270109036	0,038002283
910942	NEC8	urogenital_system	testis	-2,207108379	0,11001832
1240154	IHH-4	thyroid	thyroid	-2,032090618	0,131061236
906795	8305C	thyroid	thyroid	-1,68035304	0,18630819
924238	K5	thyroid	thyroid	-1,312904314	0,269037551
906828	CAL-62	thyroid	thyroid	-1,129067344	0,323334676
1290722	ASH-3	thyroid	thyroid	-0,849823941	0,427490189
924102	8505C	thyroid	thyroid	-0,79875015	0,449890909
930299	TT	thyroid	thyroid	-0,727032077	0,483341383
924104	BCPAP	thyroid	thyroid	-0,597689525	0,550081118

1240223	TT2609-C02	thyroid	thyroid	-0,176622889	0,83809578
906864	FTC-133	thyroid	thyroid	0,014620444	1,014727845
924151	HTC-C3	thyroid	thyroid	0,144538708	1,155506421
910568	CGTH-W-1	thyroid	thyroid	0,224204425	1,251326796
906696	BHT-101	thyroid	thyroid	0,396758938	1,486997428
1240215	SKN	urogenital_system	uterus	-0,624654626	0,535446326
908127	MES-SA	urogenital_system	uterus	0,858728283	2,360157332

Screening IBL-302 across a panel of 16 neuroblastoma cell lines

COSMIC	Cell_line	tissue	tissue2	IBL-302	IBL-302 IC50
910780	TGW	neuroblastoma	neuroblastoma	-4,30443	0,013508583
949178	NB14	neuroblastoma	neuroblastoma	-4,14914	0,01577798
949172	NB12	neuroblastoma	neuroblastoma	-3,5086	0,029938799
717431	SK-N-SH	neuroblastoma	neuroblastoma	-3,0848	0,045739181
906820	CHP-212	neuroblastoma	neuroblastoma	-3,03868	0,047898073
949174	NB7	neuroblastoma	neuroblastoma	-2,94588	0,05255579
753616	BE2-M17	neuroblastoma	neuroblastoma	-2,44057	0,087111184
908447	NH-12	neuroblastoma	neuroblastoma	-2,40332	0,090417269
949177	NB13	neuroblastoma	neuroblastoma	-2,39882	0,090825064
906875	GOTO	neuroblastoma	neuroblastoma	-2,36045	0,094377744
907170	IMR-5	neuroblastoma	neuroblastoma	-2,35987	0,094432499
753618	KELLY	neuroblastoma	neuroblastoma	-2,2075	0,109975243
1240181	NB(TU)1-10	neuroblastoma	neuroblastoma	-1,91249	0,147712125
688087	SK-N-FI	neuroblastoma	neuroblastoma	-1,78474	0,167840694
949179	NB1	neuroblastoma	neuroblastoma	-1,48075	0,227467024
724828	SK-N-AS	neuroblastoma	neuroblastoma	-0,12902	0,878956386
