# Supplementary material for: Anti‐tumor effects of PIM/PI3K/mTOR triple kinase inhibitor IBL‐302 in neuroblastoma
Source: EMBO Mol Med. 2019 Jul 16;11(8):e10058. doi: 10.15252/emmm.201810058 (PMC6685085; doi:10.15252/emmm.201810058)
Supplement: Supplementary file 2 — Expanded View Figures PDF [file EMMM-11-e10058-s002.pdf]

Expanded View Figures

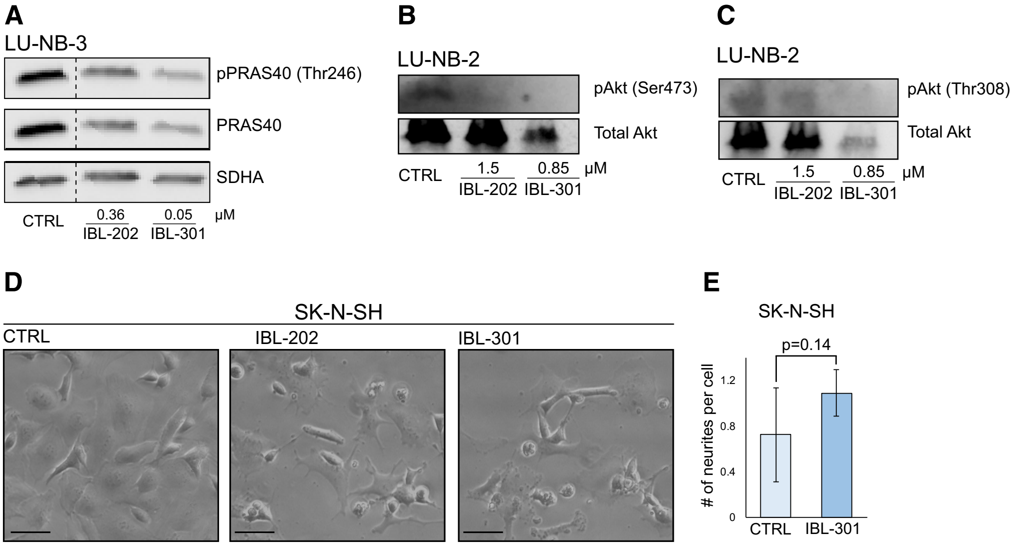

Figure EV1. Multikinase inhibitors target downstream signaling pathways.

A–C Western blot analyses of downstream signaling protein pPRAS40 (A), pAkt(Ser473) (B), and pAkt(Thr308) (C) expression in LU-NB-3 and LU-NB-2 PDX cells after treatment with IBL-202 or IBL-301 at indicated concentrations for 48 h. PRAS40, total Akt, and SDHA levels were used as loading controls.

D Brightfield photomicrographs of SK-N-SH cells treated with 0.36 μM IBL-202 or 0.05 μM IBL-301 for 48 h. Scale bars represent 100 μm.

E Quantification of neurite outgrowth presented as number of neurites/cell in SK-N-SH cells treated with 0.05 μM IBL-301. Representative areas (n = 4 and n = 7 for CTRL and IBL-301, respectively) were used, and n = 249 and n = 245 cells/condition for CTRL and IBL-301, respectively, were counted. Values are reported as mean ± SEM. Statistical significance was determined by two-sided Student's t-test. P = 0.14.

Source data are available online for this figure.

 © 2019 The Authors

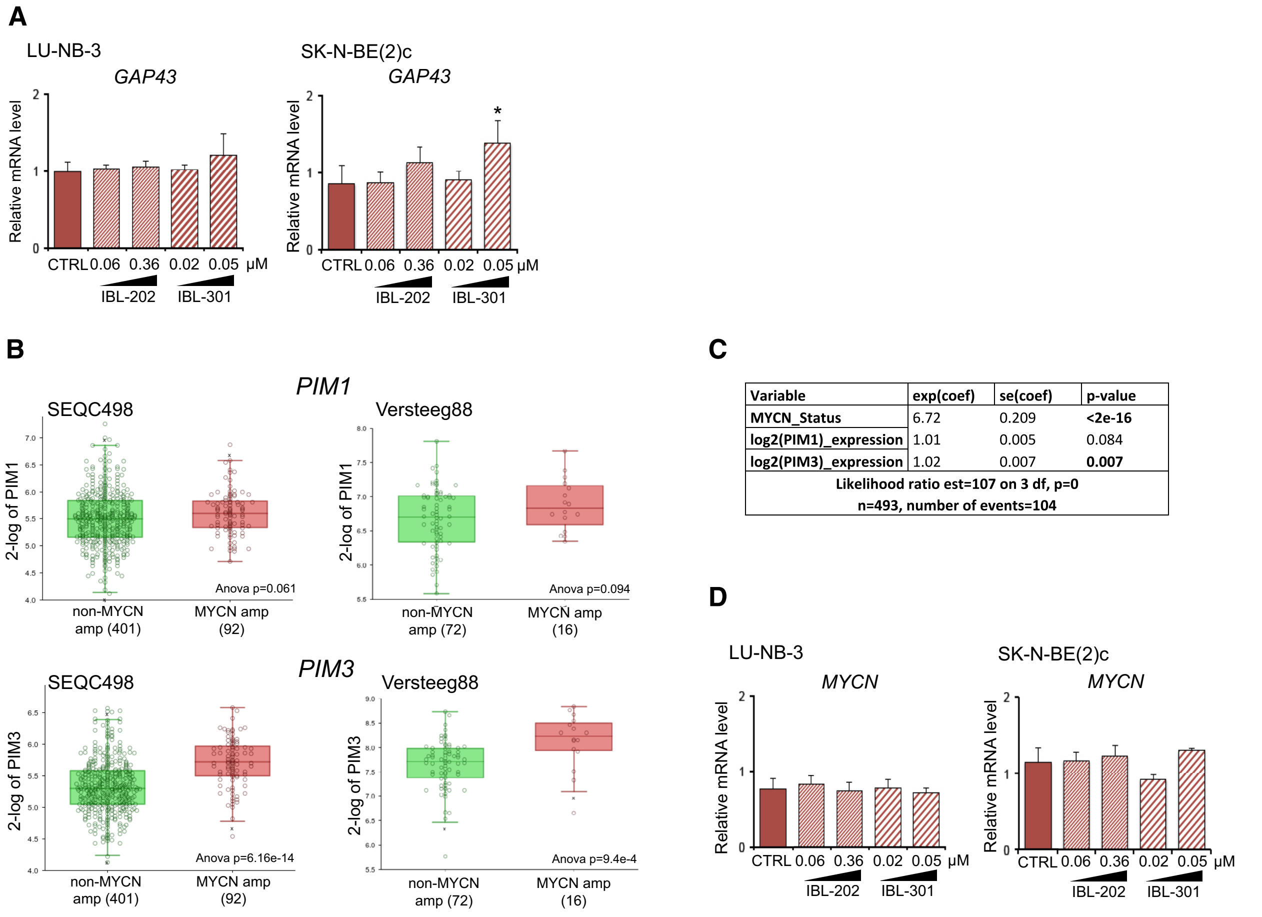

Variable	exp(coef)	se(coef)	p-value
MYCN_Status	6.72	0.209	**<2e-16**
log2(PIM1)_expression	1.01	0.005	0.084
log2(PIM3)_expression	1.02	0.007	**0.007**
Likelihood ratio est=107 on 3 df, p=0			
n=493, number of events=104			

Figure EV2. PIM3 is expressed at higher levels in MYCN-amplified tumors.

A Relative mRNA expression levels of *GAP43* in LU-NB-3 and SK-N-BE(2)c cells treated with IBL-202 or IBL-301 at indicated concentrations for 48 h as determined by qRT–PCR. Mean values from three biologically independent experiments. Error bars represent SEM. Statistical significance was determined by one-way ANOVA. **P* = 0.013.

B *PIM1* and *PIM3* expression in non-*MYCN*-amplified vs. *MYCN*-amplified tumors in publicly available datasets SEQC498 (left panels) and Versteeg88 (right panels). Horizontal bands are the median line; the upper part of the box is the 1st quartile and the lower box is the 3rd quartile; the error bars are the maximum and minimum values excluding outliers; the X symbols are mean values.

C Determination of *PIM1* and *PIM3 MYCN* dependence through multivariate cox regression analysis in publicly available dataset SEQC498. The text in the lowest row in this Table is random and not everything is included.

D Relative mRNA expression levels of *MYCN* in LU-NB-3 and SK-N-BE(2)c cells treated with IBL-202 or IBL-301 at indicated concentrations for 48 h as determined by qRT–PCR. Mean values from three biologically independent experiments. Error bars represent SEM. Statistical significance was determined by one-way ANOVA. No asterisk indicates no significance.

Figure EV3. Combined targeting of PIM, PI3K, and mTOR is more efficient than single-target treatment.

A–C LU-NB-3 PDX cells treated with PI-103 (PI3K inhibitor, Number of replicates; A), PP242 (mTORC1/2 inhibitor; B), or AZD1208 (PIM inhibitor; C) at indicated concentrations. Cell viability determined by CellTiter-Glo. Values are reported as mean ± SEM (*n* = 2).

D LU-NB-3 PDX cells treated with 0.13 µM PI-103, 10.2 µM PP242, 0.1 µM AZD1208, or the combination of all three inhibitors. Cell death determined by Annexin V assay and flow cytometry.

E Quantification of live and dead cells from (D). Live cells = PI negative, dead cells = PI positive. Mean values from two biologically independent experiments. Error bars represent SEM.

F Treatment of SK-N-AS or SK-N-FI neuroblastoma cells with 50–500 nM IBL-302, 100–1,000 nM PI-103, or 10–1,000 nM dactolisib. Cell viability determined by CellTiter-Glo. Values are reported as mean ± SEM (*n* = 3).

G Tumor size of SK-N-AS xenograft mice treated with IBL-302 or dactolisib for indicated amount of days (until first mouse in each group reached maximum tolerated tumor volume). Mice were randomly allocated to either of the three groups (*n* = 7, CTRL; *n* = 8, IBL-302; *n* = 8, dactolisib). Values are reported as mean ± SEM. Statistical analysis was performed at day 11 when all mice included in the study were alive; ANOVA *P* = 0.5518.

© 2019 The Authors

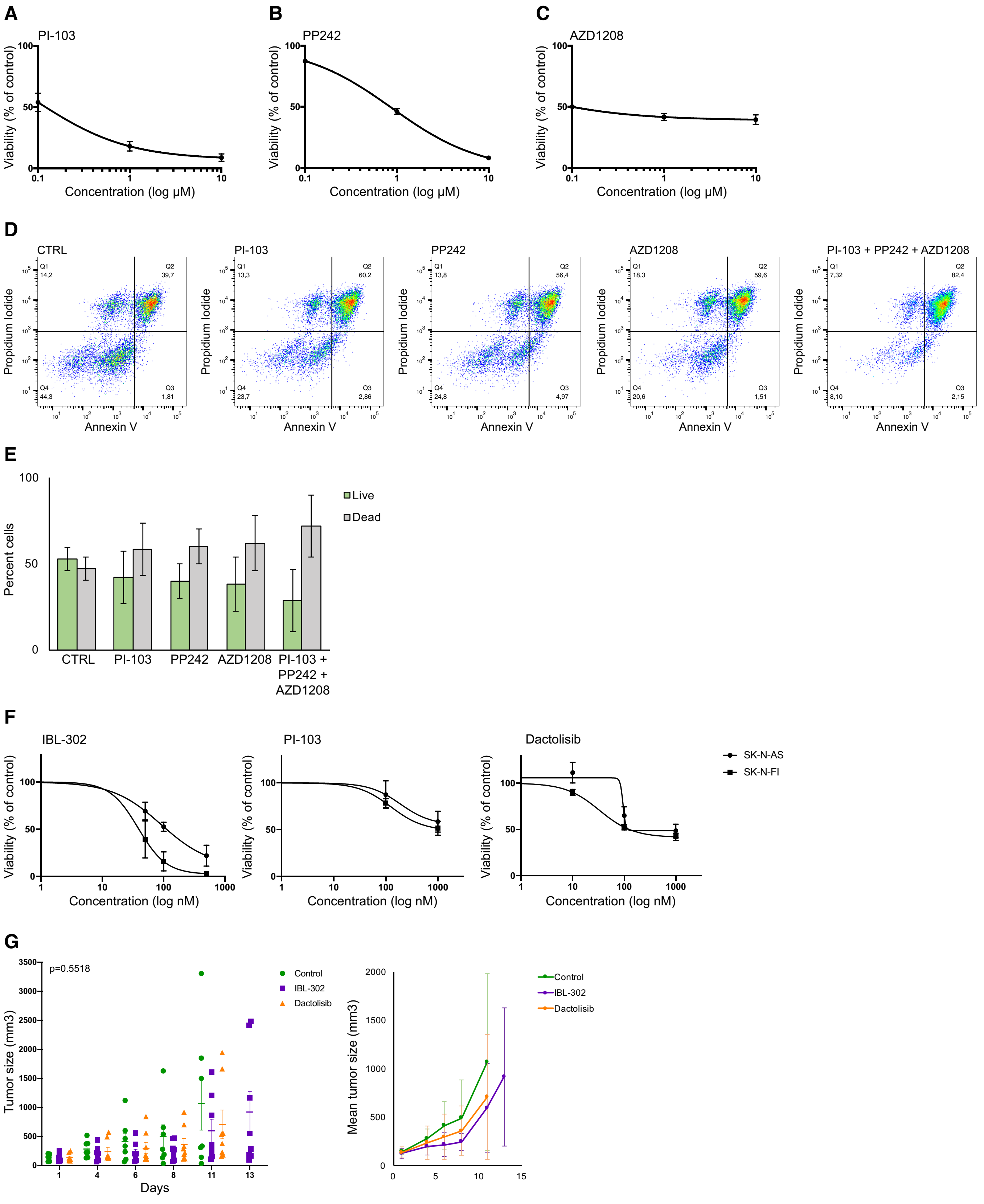

Figure EV3.

© 2019 The Authors

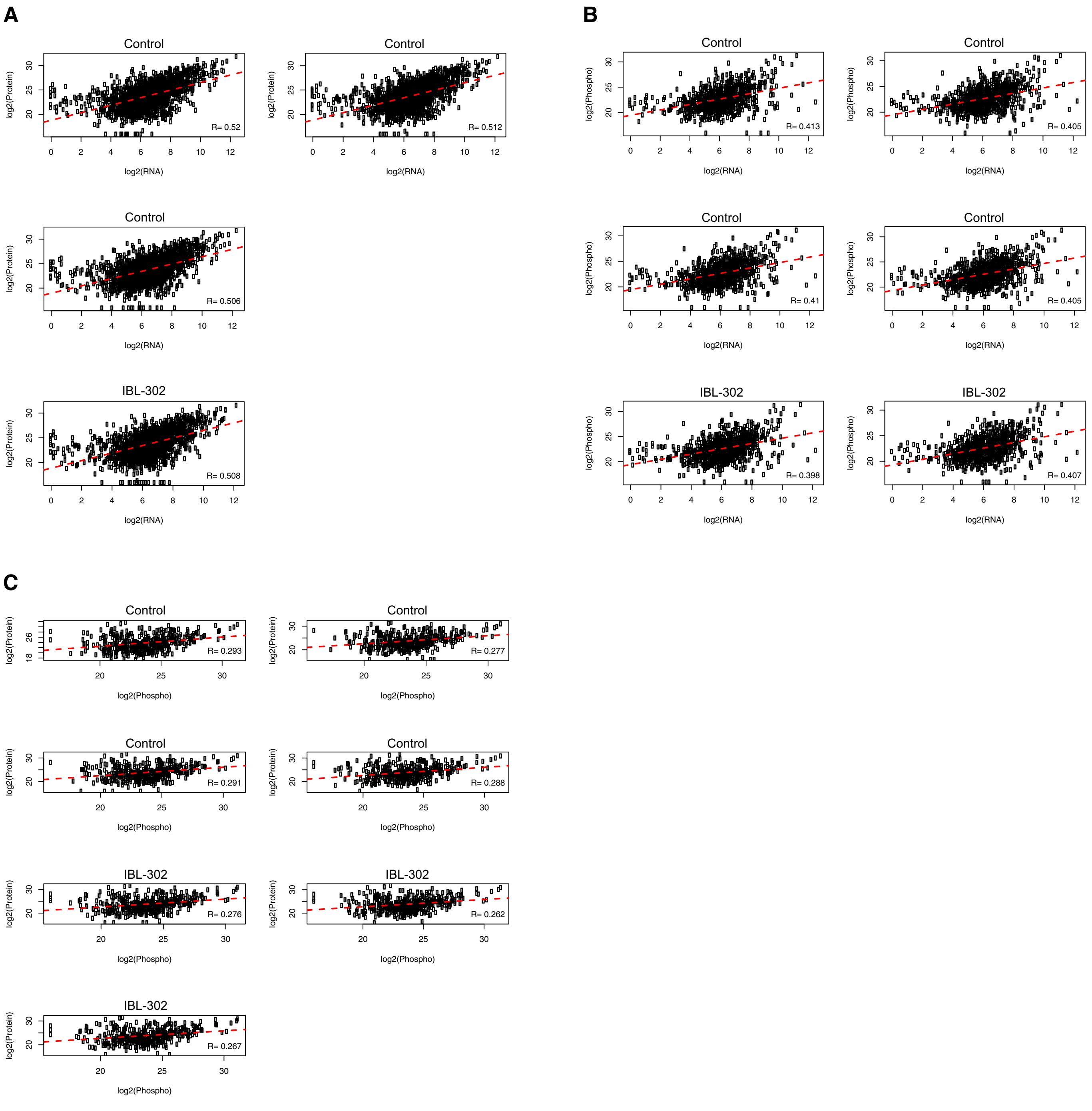

Figure EV4. Correlations between RNA, protein, and phospho-protein expression.

A–C Cross-platform sample correlation of expression levels between RNAseq and mass spectrometry (A), RNAseq and mass spectrometry phospho-proteome (B), and mass spectrometry proteome and mass spectrometry phospho-proteome (C) data. Pearson's correlation coefficients are displayed.
