# Supplementary figures and images for: Anti‐tumor effects of PIM/PI3K/mTOR triple kinase inhibitor IBL‐302 in neuroblastoma
Source: EMBO Mol Med. 2019 Jul 16;11(8):e10058. doi: 10.15252/emmm.201810058 (PMC6685085; doi:10.15252/emmm.201810058)

## For figure S2

pAkt (s473)

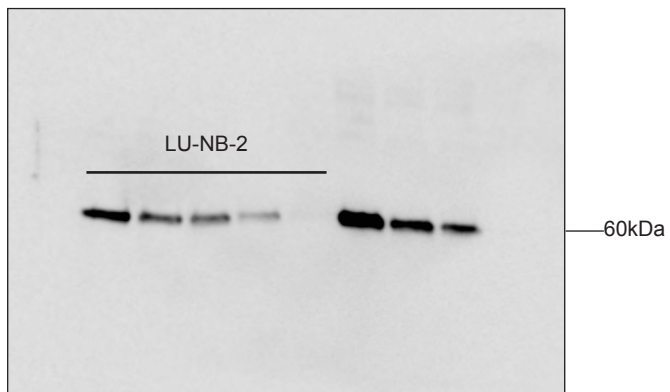

Total akt

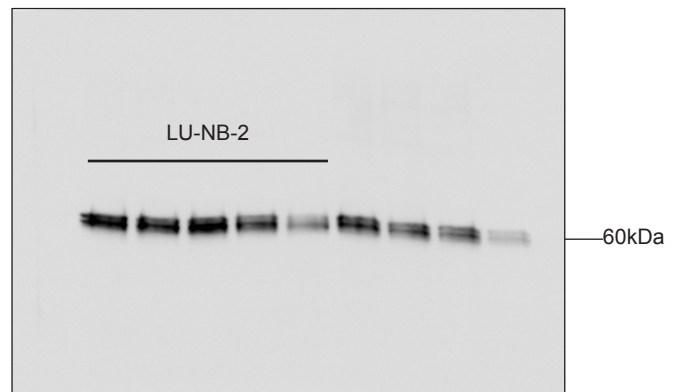

Actin

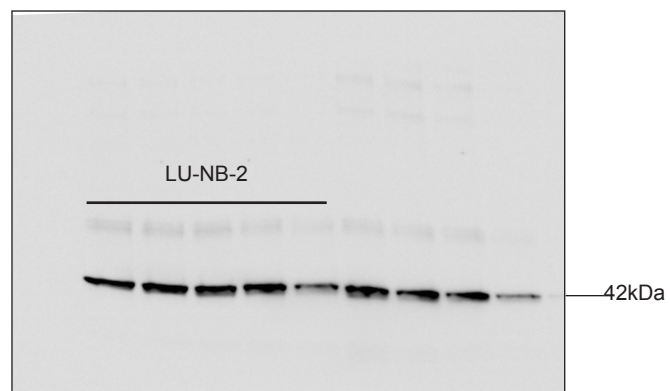

(Also used for figure 5)

pAkt (t308)

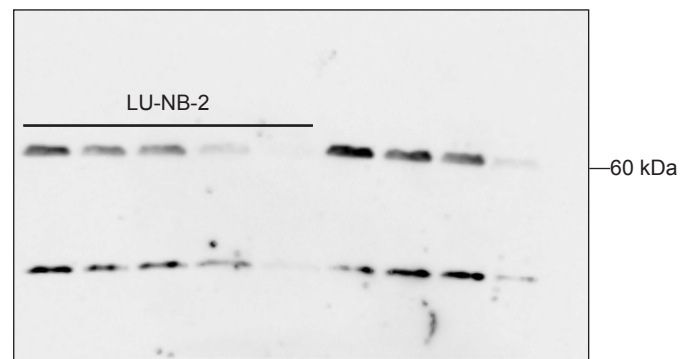

Total akt

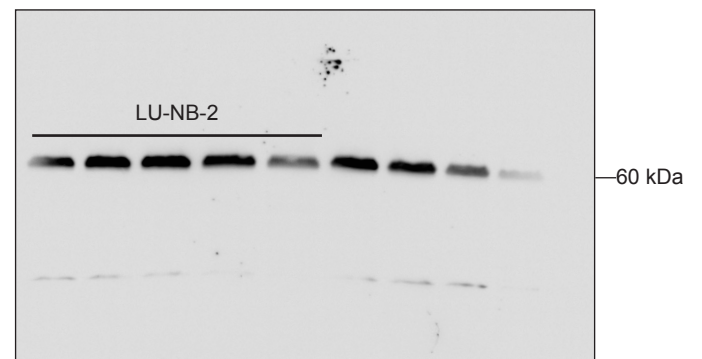

Actin

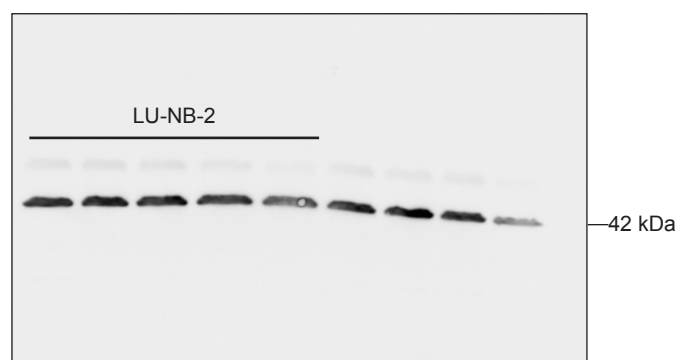

CTRL

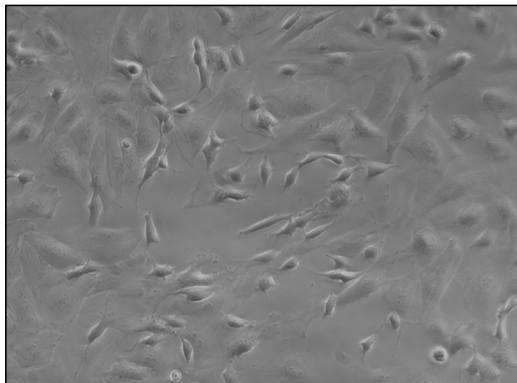

IBL-302

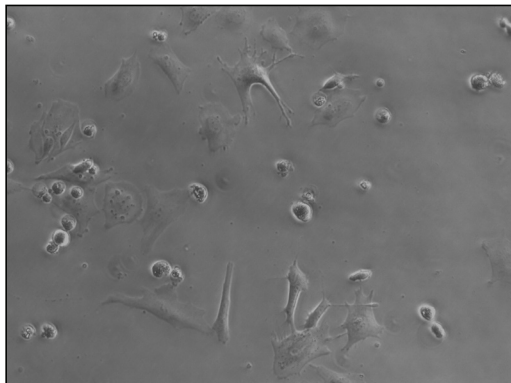

Supplement: Supplementary file 4 — Source Data for Expanded View and Appendix [file EMMM-11-e10058-s008.zip › EMM_10058_EV_appendix_source_data/Source_data_for_Appendix_Figure_S2.pdf]

For figure EV1

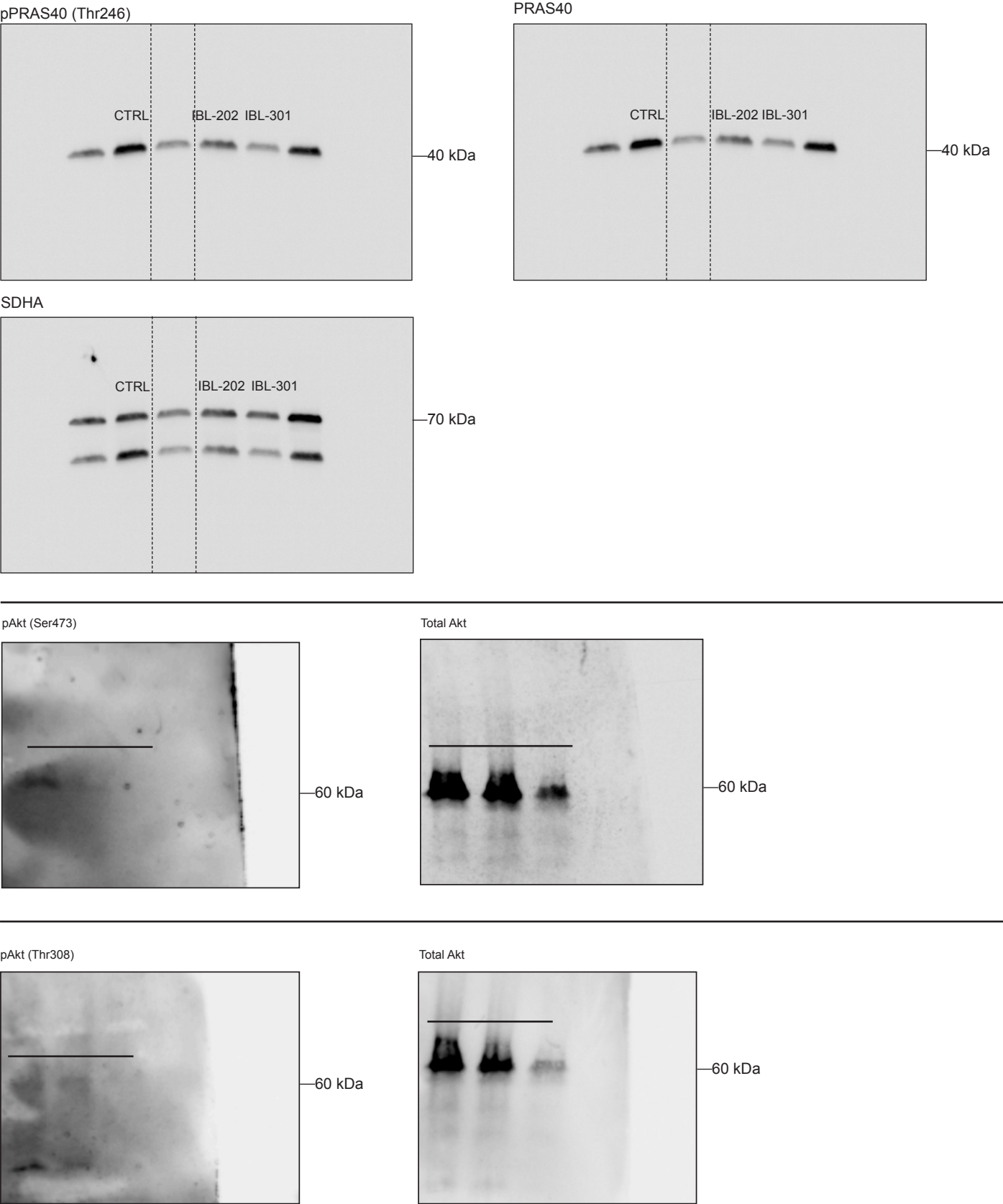

CTRL

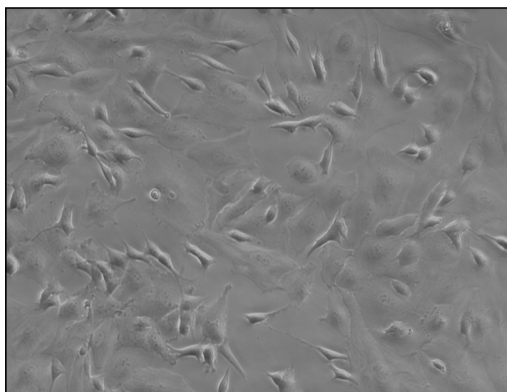

IBL-202

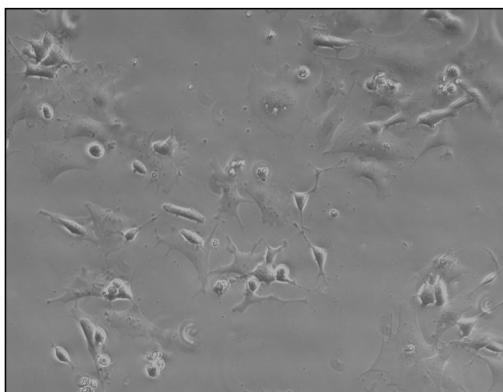

IBL-301

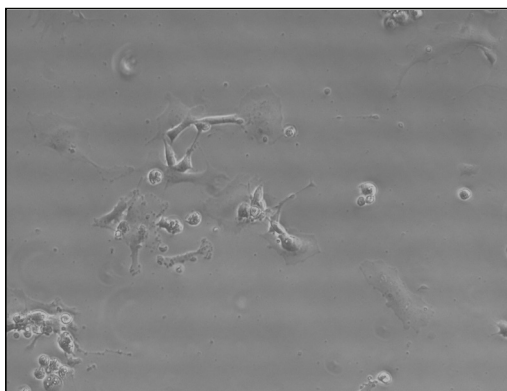

Supplement: Supplementary file 4 — Source Data for Expanded View and Appendix [file EMMM-11-e10058-s008.zip › EMM_10058_EV_appendix_source_data/Source_Data_for_EV_Figure_1.pdf]

For figure 3

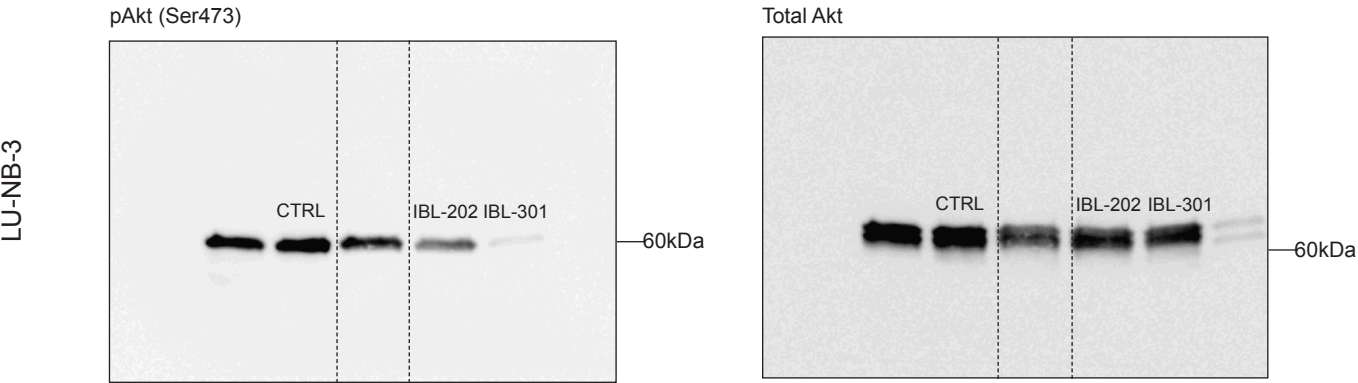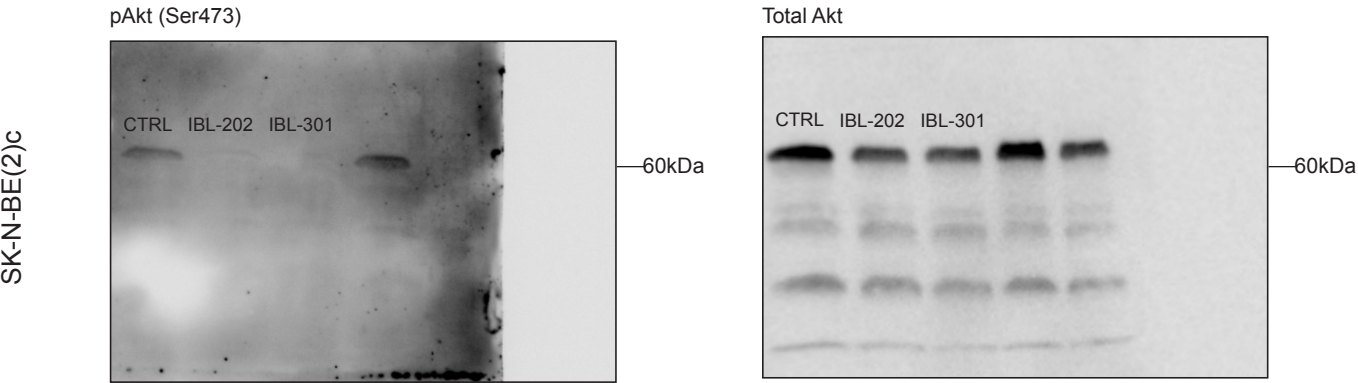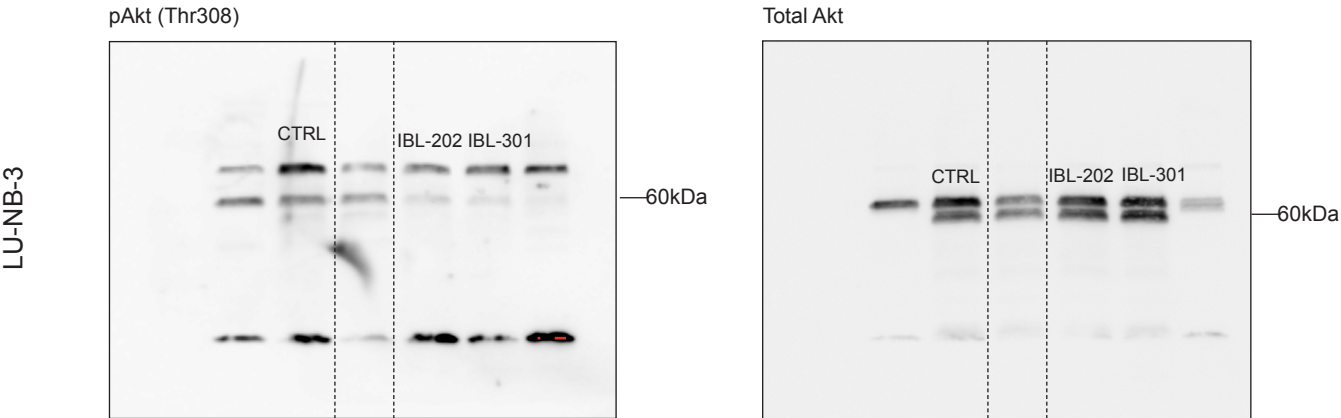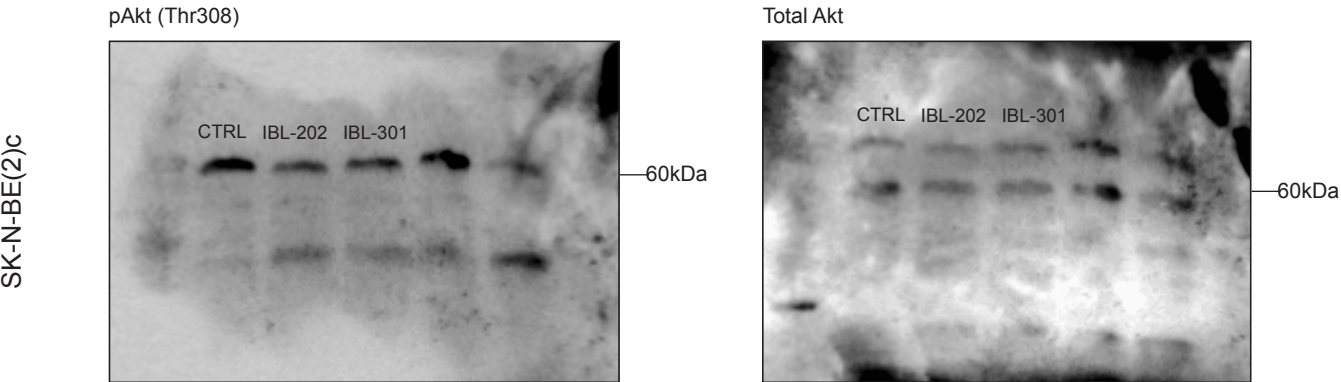

LU-NB-3

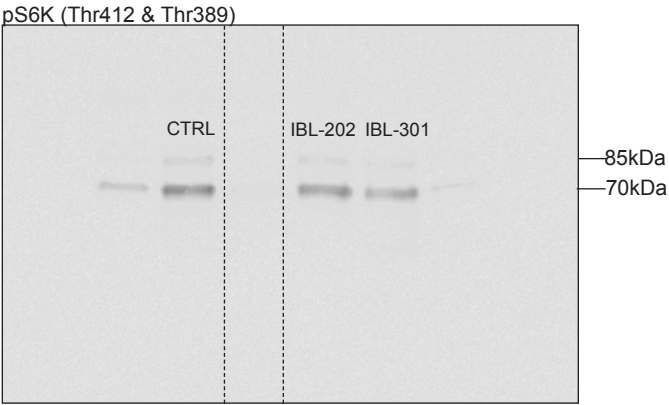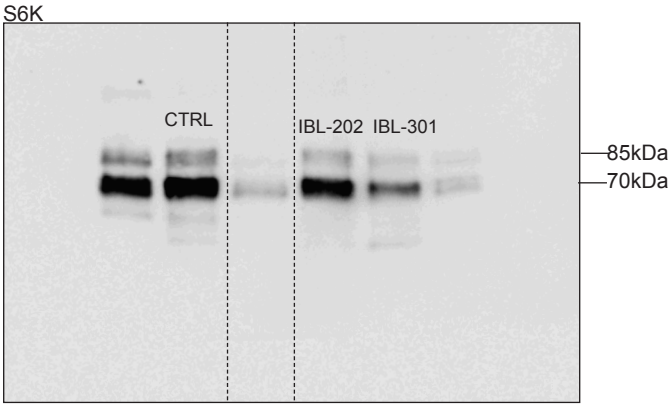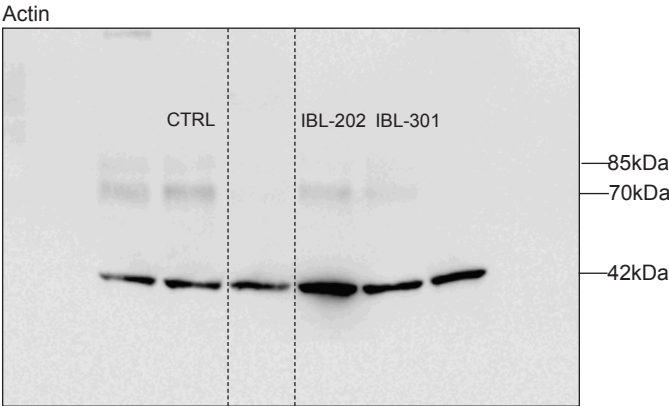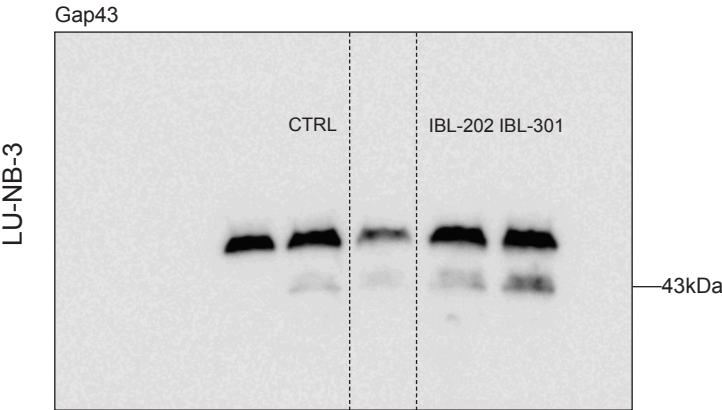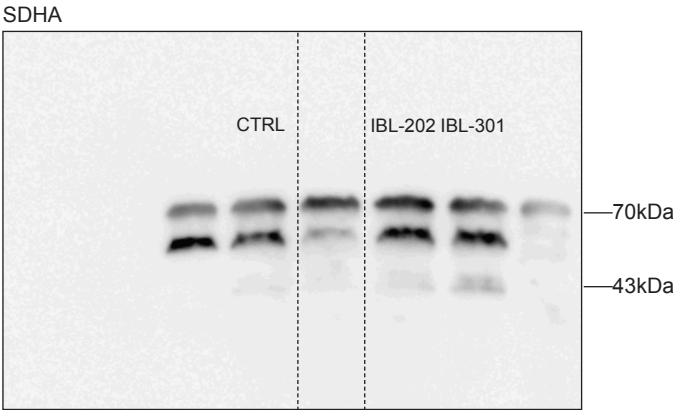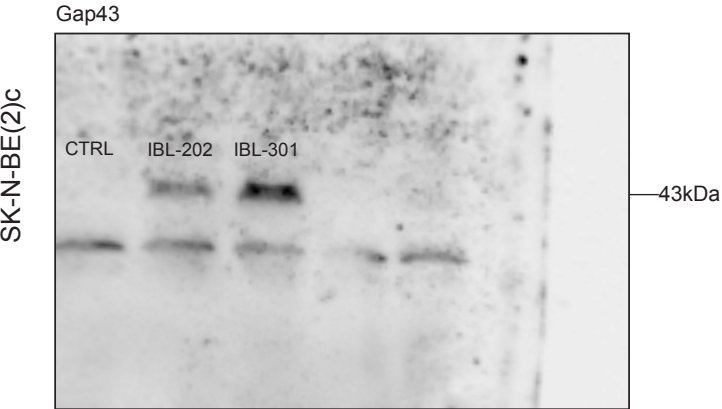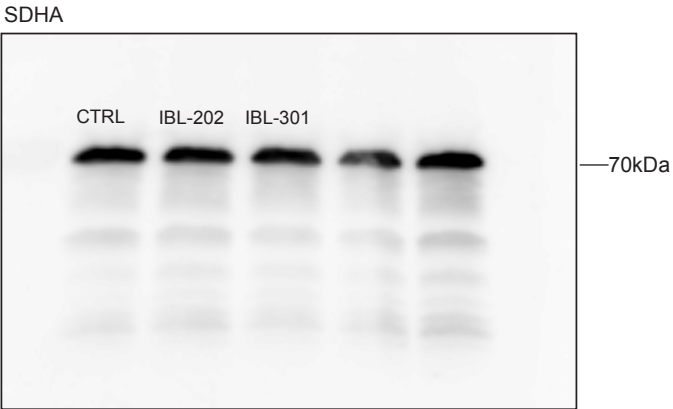

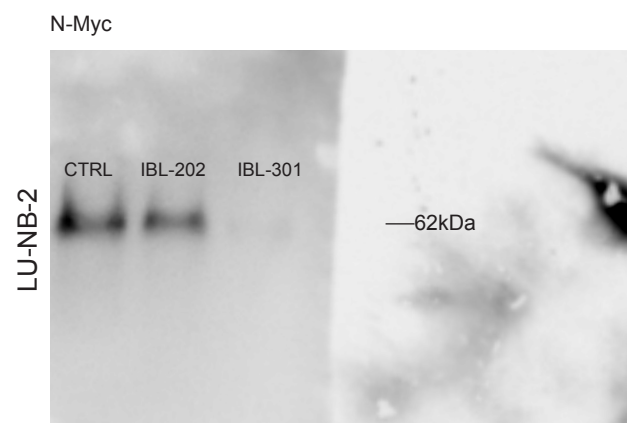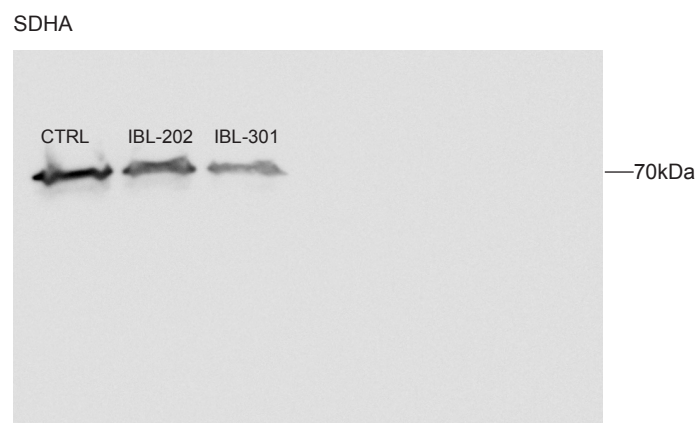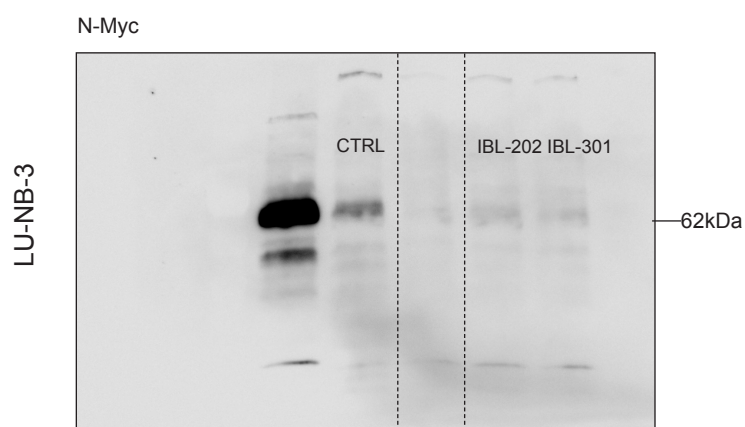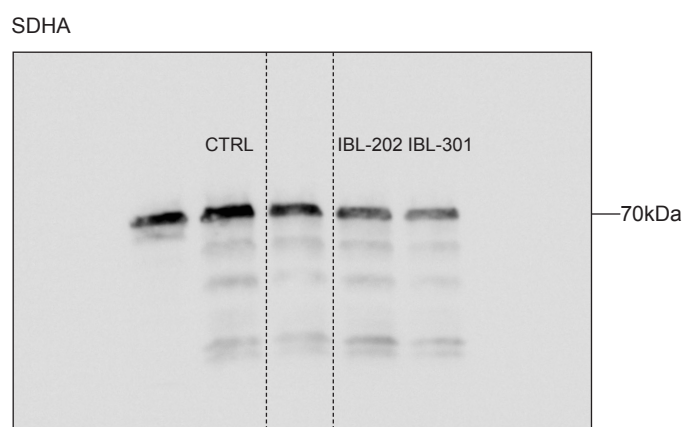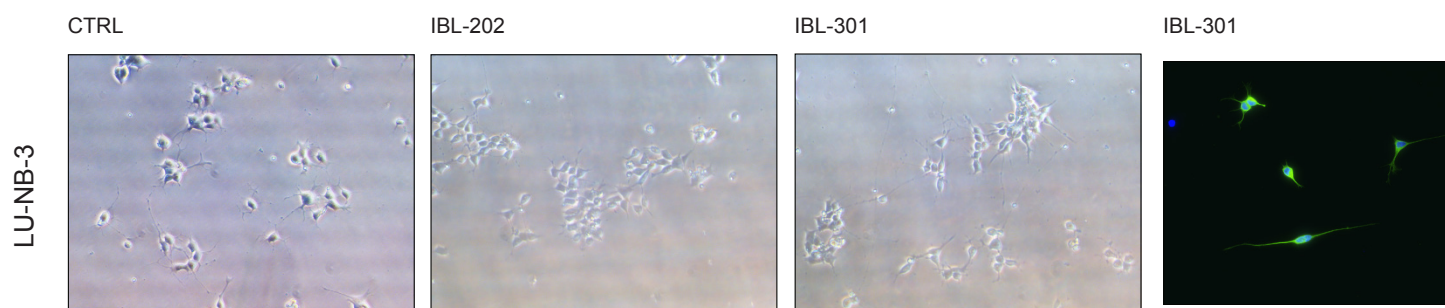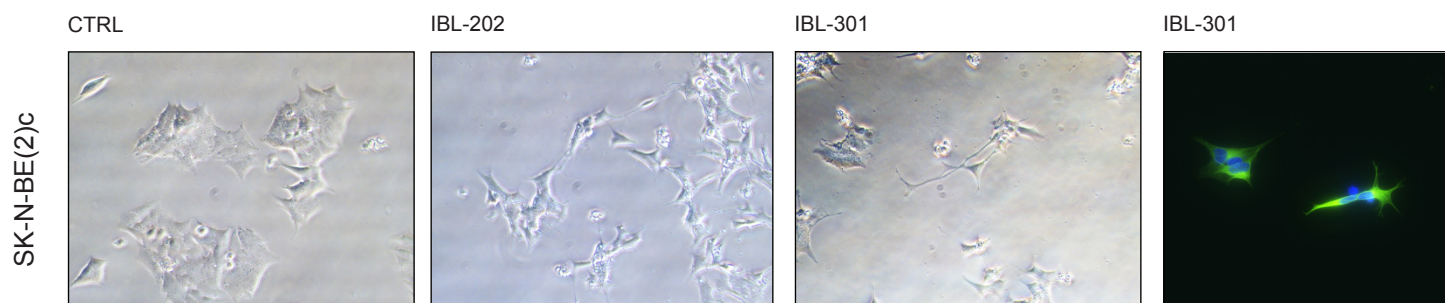

Supplement: Supplementary file 6 — Source Data for Figure 3 [file EMMM-11-e10058-s004.pdf]

For figure 4

Casp3

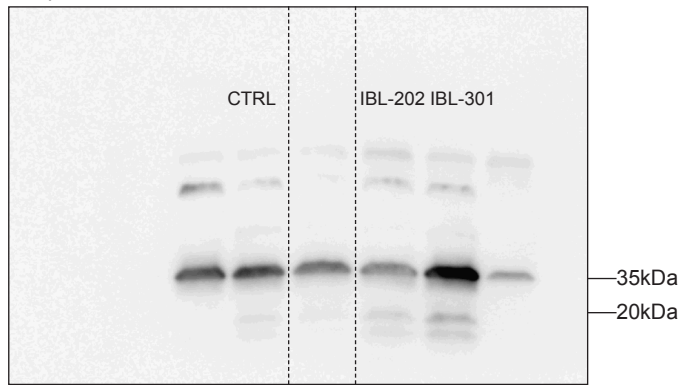

SDHA

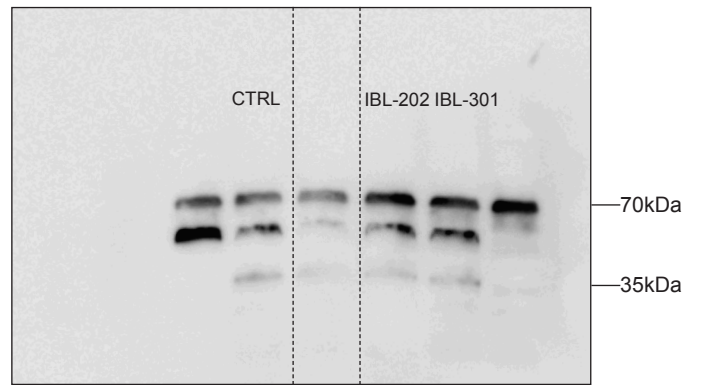

Supplement: Supplementary file 7 — Source Data for Figure 4 [file EMMM-11-e10058-s005.pdf]

For figure 5

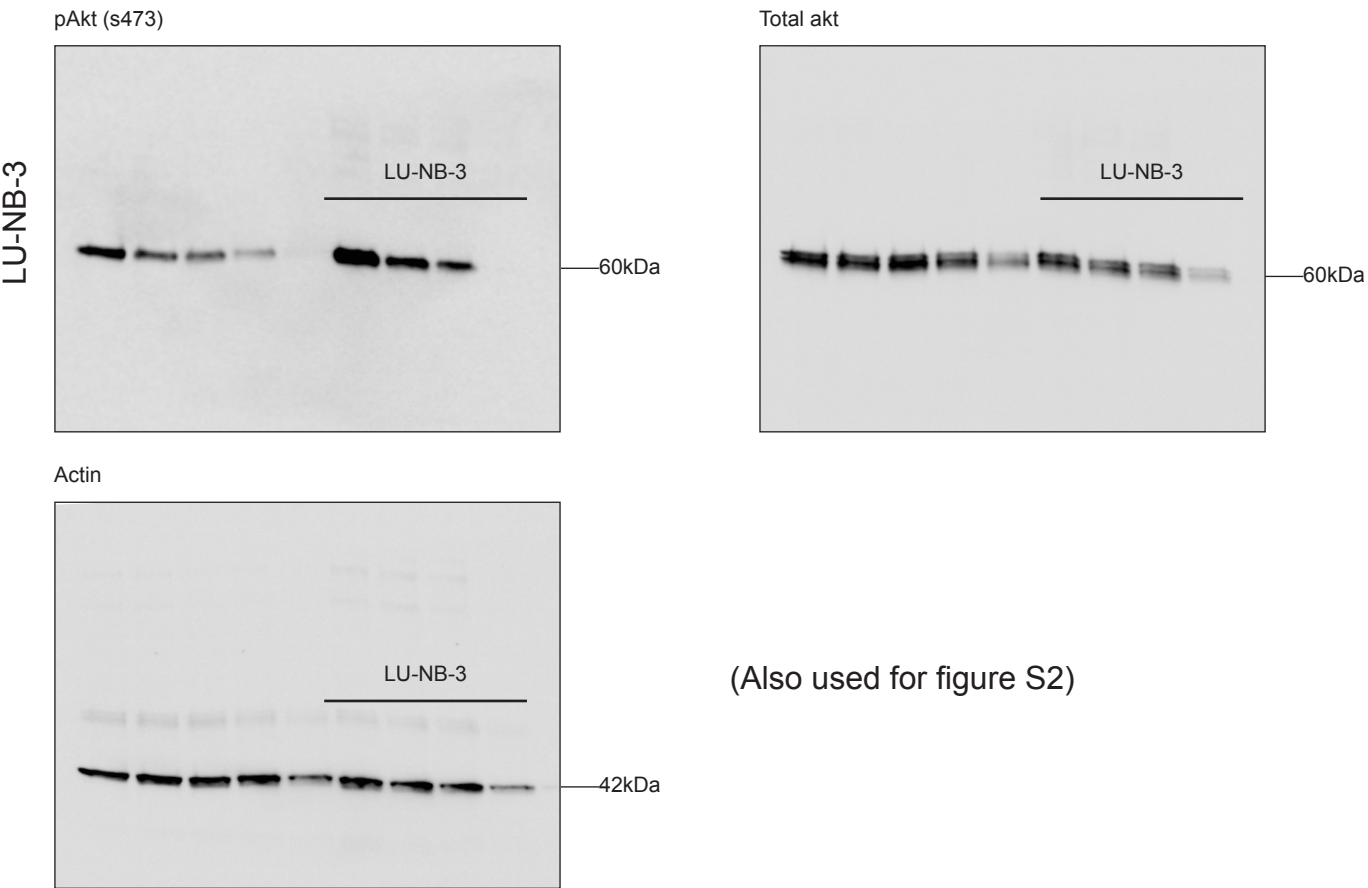

(Also used for figure S2)

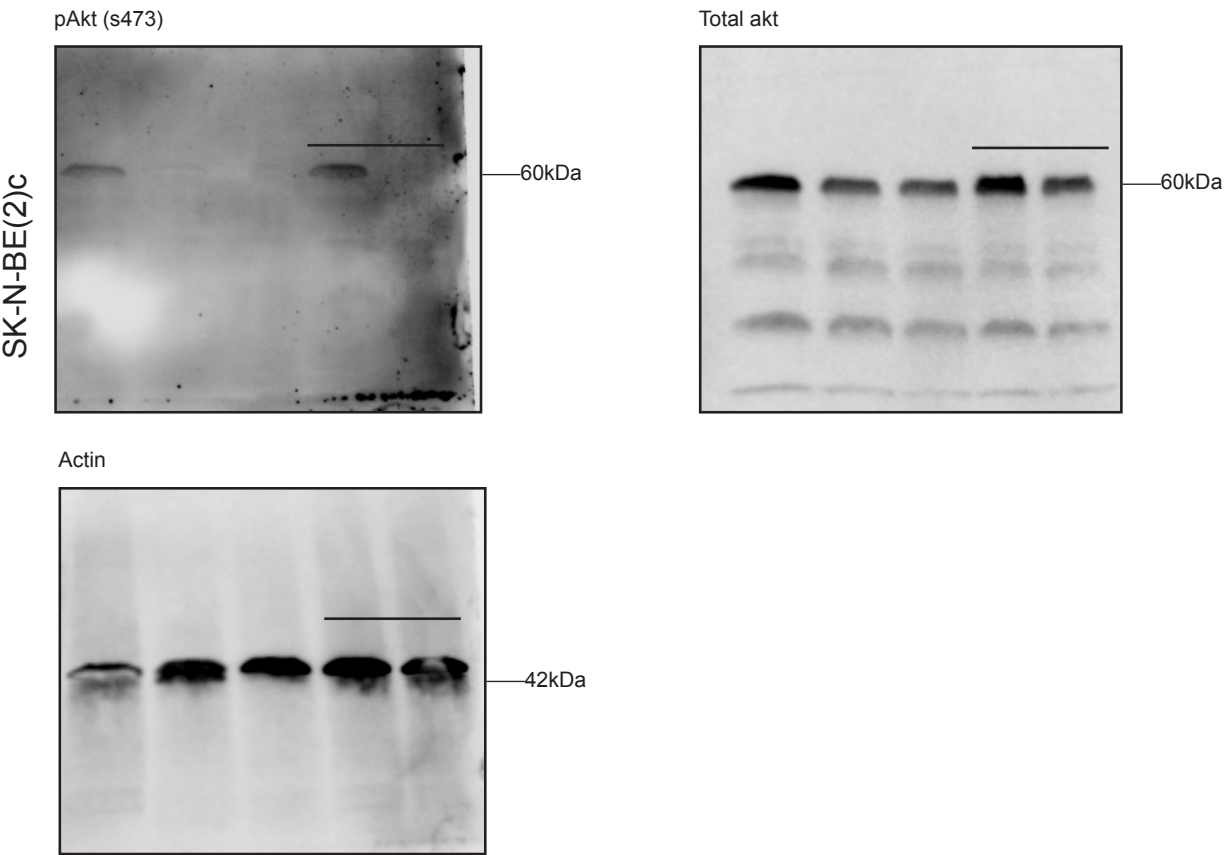

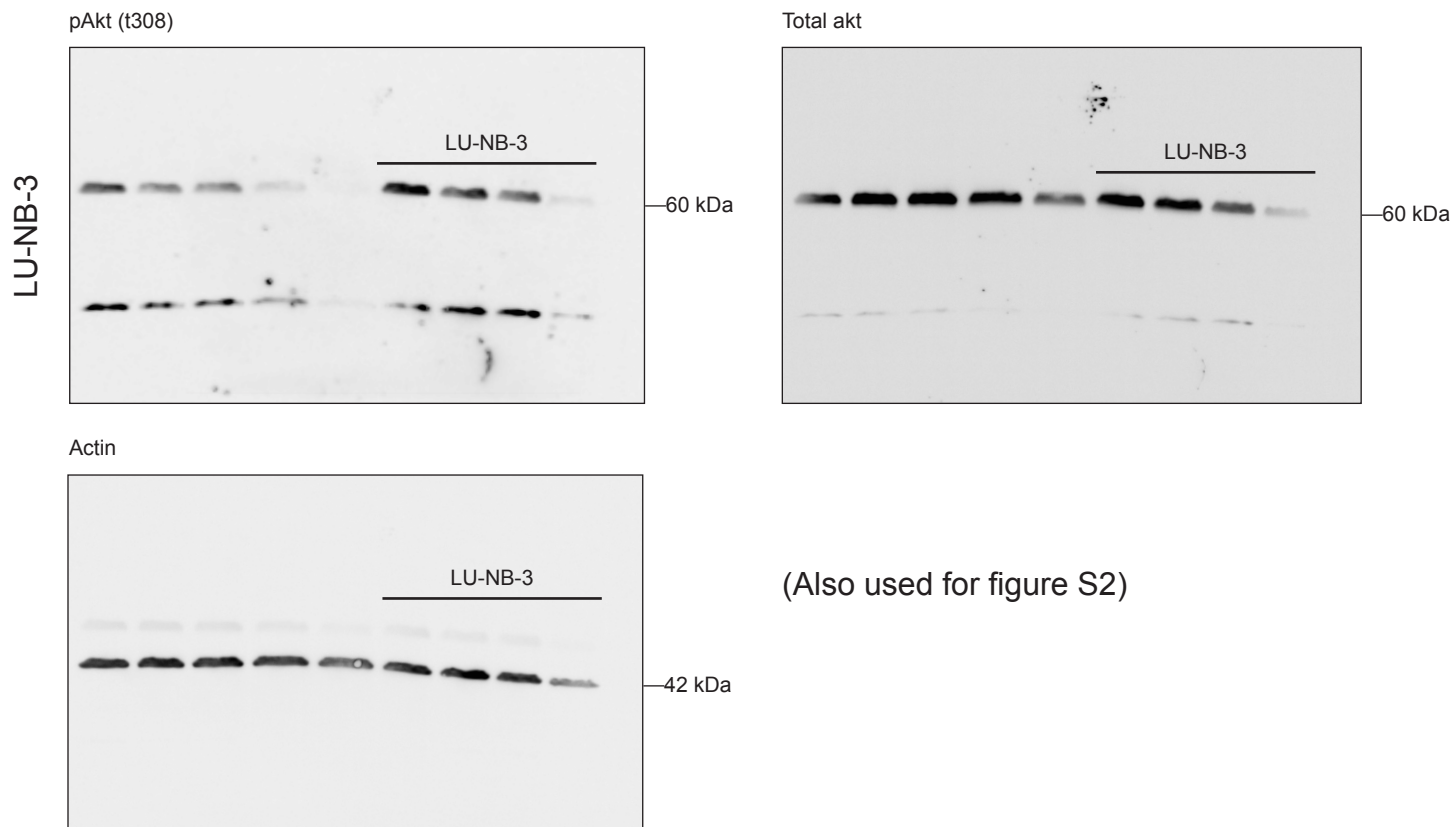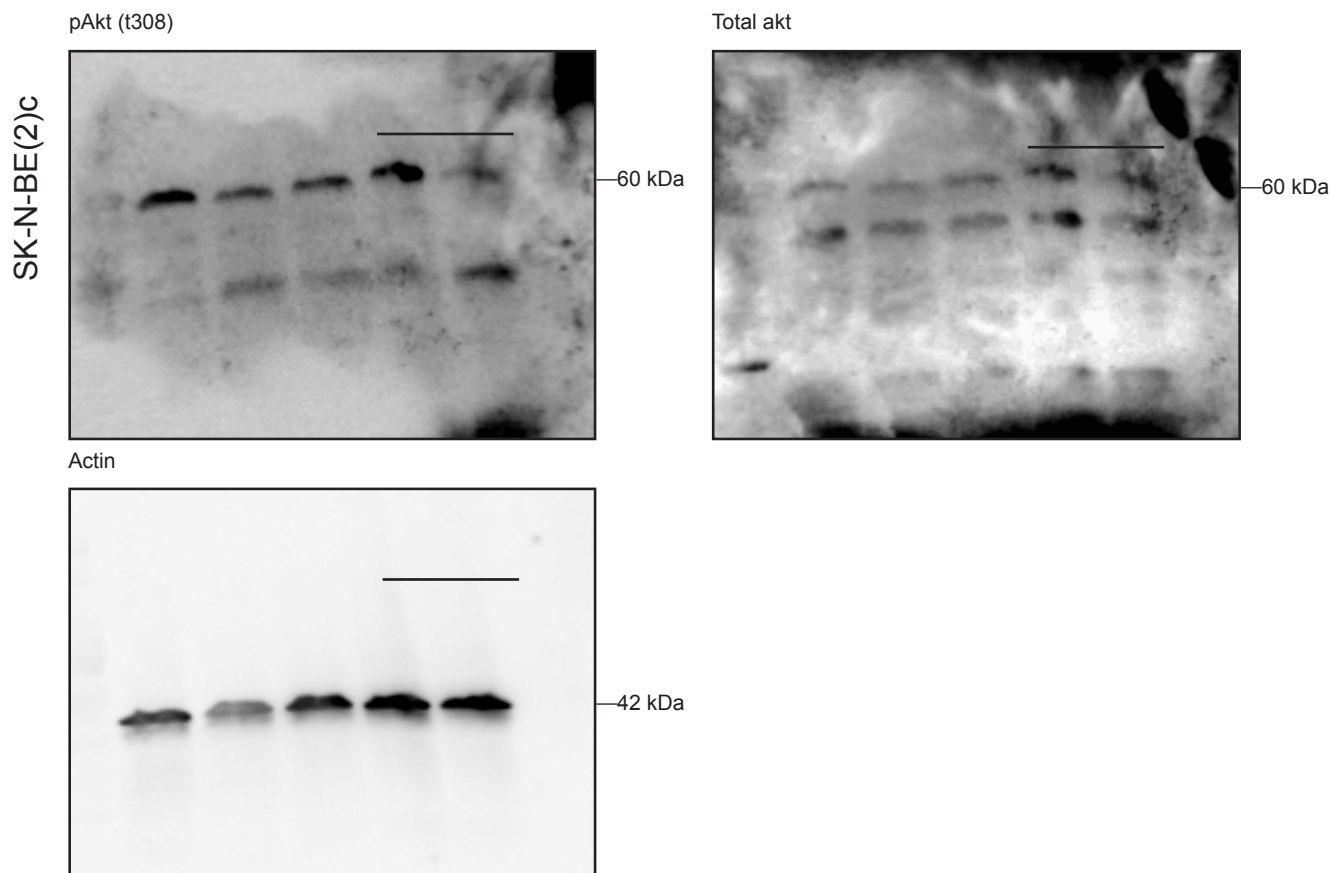

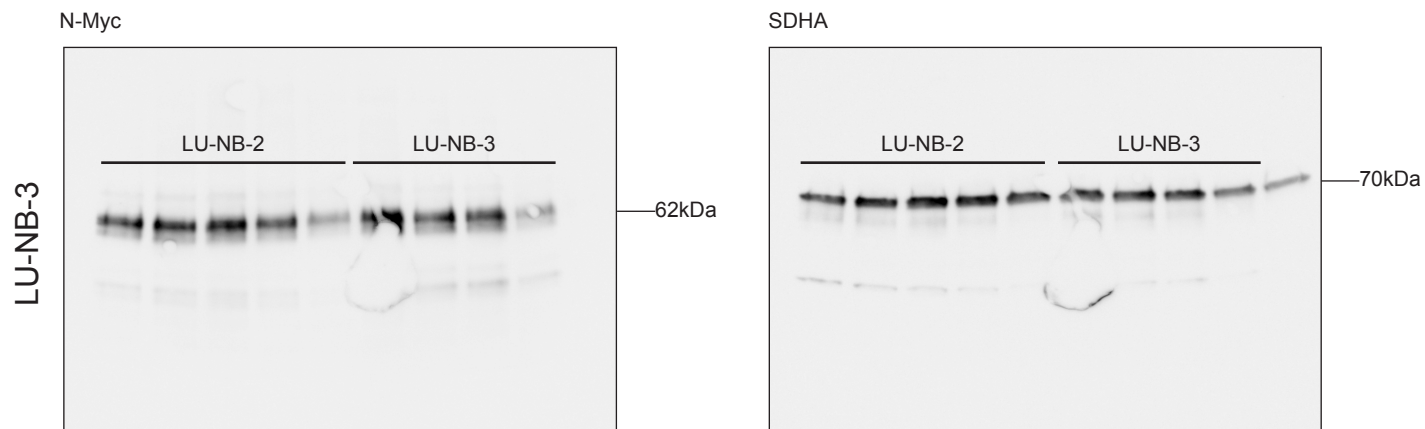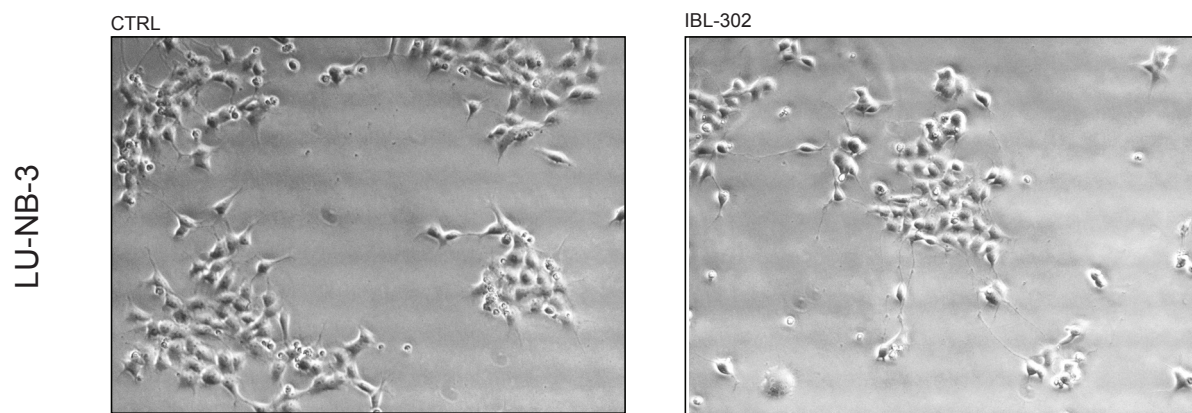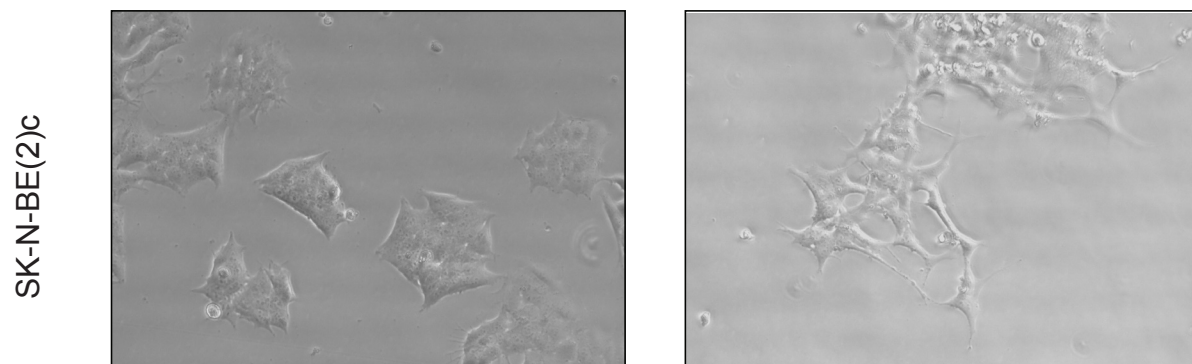

Supplement: Supplementary file 8 — Source Data for Figure 5 [file EMMM-11-e10058-s006.pdf]

For figure 6

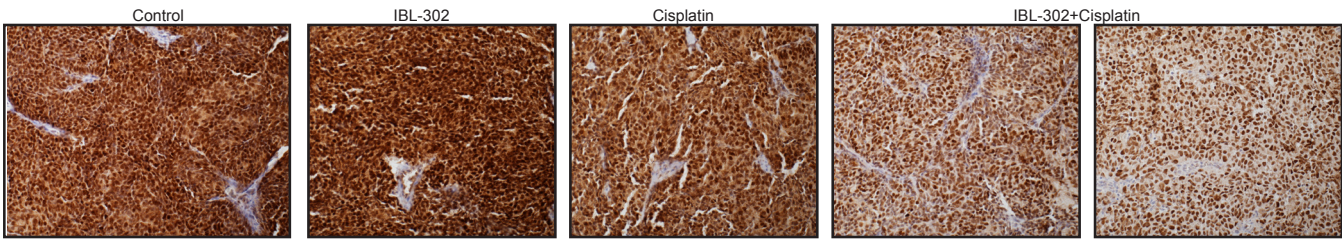

Supplement: Supplementary file 9 — Source Data for Figure 6 [file EMMM-11-e10058-s007.pdf]
